# Intestinal epithelial glucocorticoid receptor promotes chronic inflammation–associated colorectal cancer

**DOI:** 10.1172/jci.insight.151815

**Published:** 2021-12-22

**Authors:** Shuang Tang, Zhan Zhang, Robert H. Oakley, Wenling Li, Weijing He, Xiaojiang Xu, Ming Ji, Qing Xu, Liang Chen, Alicia S. Wellman, Qingguo Li, Leping Li, Jian-Liang Li, Xinxiang Li, John A. Cidlowski, Xiaoling Li

**Affiliations:** 1 Signal Transduction Laboratory, National Institute of Environmental Health Sciences, NIH, Durham, North Carolina, USA.; 2 Cancer Institute and Department of Nuclear Medicine, Fudan University Shanghai Cancer Center, Shanghai, China.; 3 Central for Global Health, School of Public Health, Nanjing Medical University, Nanjing, Jiangsu, China.; 4 Biostatistics and Computational Biology Branch, National Institute of Environmental Health Sciences, NIH, Durham, North Carolina, USA.; 5 Department of Colorectal Surgery, Fudan University Shanghai Cancer Center, Department of Oncology, Shanghai Medical College, Fudan University, Shanghai, China.; 6 Integrative Bioinformatics Group, National Institute of Environmental Health Sciences, NIH, Durham, North Carolina, USA.; 7 Department of Urology, Zhongnan Hospital of Wuhan University, Wuhan, Hubei, China.

**Keywords:** Gastroenterology, Oncology, Colorectal cancer, Inflammatory bowel disease, Innate immunity

## Abstract

Synthetic immunosuppressive glucocorticoids (GCs) are widely used to control inflammatory bowel disease (IBD). However, the impact of GC signaling on intestinal tumorigenesis remains controversial. Here, we report that intestinal epithelial GC receptor (GR), but not whole intestinal tissue GR, promoted chronic intestinal inflammation-associated colorectal cancer in both humans and mice. In patients with colorectal cancer, GR was enriched in intestinal epithelial cells and high epithelial cell GR levels were associated with poor prognosis. Consistently, intestinal epithelium–specific deletion of GR (GR iKO) in mice increased macrophage infiltration, improved tissue recovery, and enhanced antitumor response in a chronic inflammation–associated colorectal cancer model. Consequently, GR iKO mice developed fewer and less advanced tumors than control mice. Furthermore, oral GC administration in the early phase of tissue injury delayed recovery and accelerated the formation of aggressive colorectal cancers. Our study reveals that intestinal epithelial GR signaling repressed acute colitis but promoted chronic inflammation–associated colorectal cancer. Our study suggests that colorectal epithelial GR could serve as a predictive marker for colorectal cancer risk and prognosis. Our findings further suggest that, although synthetic GC treatment for IBD should be used with caution, there is a therapeutic window for GC therapy during colorectal cancer development in immunocompetent patients.

## Graphical abstract

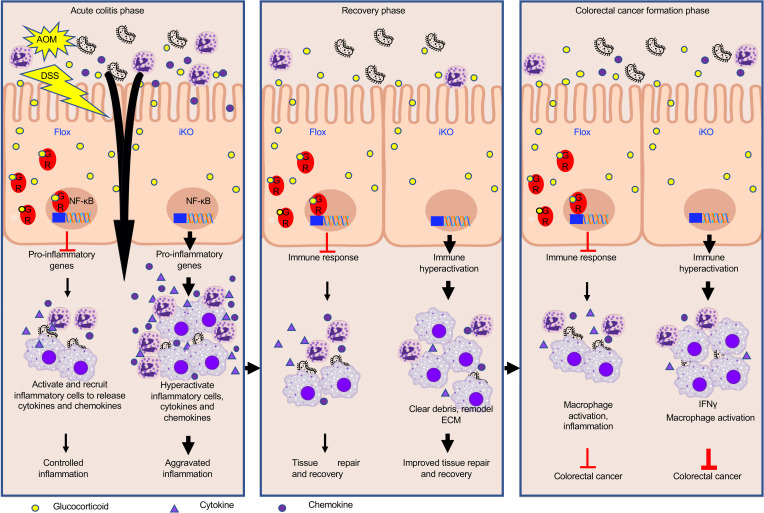

## Introduction

Glucocorticoids (GCs) are stress-induced steroid hormones that regulate diverse metabolic pathways and the immune system. This class of hormones functions by binding GC receptor (GR), a ligand-activated transcription factor that regulates a diverse array of genes to maintain cellular homeostasis (1).

GC signaling is an integral part of the signaling network critical for the intestinal stress response and tissue homeostasis (2). Physiological, environmental, and emotional stress induces the release of GCs from the adrenal gland as well as from the intestinal epithelium; these GCs are critical in modulation of immune suppression and antiinflammation of the intestinal mucosa (3, 4). Dysregulation of intestinal stress response disrupts intestinal tissue homeostasis, resulting in the development of inflammatory bowel disease (IBD), a class of chronic intestinal mucosa immunopathologies that includes ulcerative colitis and Crohn’s disease (5). Chronic inflammation in the intestine is also a critical component of tumor progression (6, 7), and consistently, patients with IBD have been shown to be at significantly increased risk of colorectal cancer (8). The potent antiinflammatory activities of GCs lead to their wide prescription for the treatment of IBD. However, the exact role of GC signaling in the regulation of intestinal tissue homeostasis, especially colorectal tumorigenesis, remains unclear.

The impact of GC signaling on cancer progression varies depending on cancer type and genetic background, despite of the prevalent use of synthetic GC in clinical oncology. For instance, synthetic GCs are often used to treat hematologic malignancies due to their ability to inhibit the proinflammatory transcription factor NF-κB, enhance proapoptotic genes, and induce cell cycle arrest (9). In nonhematologic cancers, GCs are commonly used as supportive care comedication for patients with cancer receiving standard therapies due to their antiemetic, antiinflammatory, and energy/appetite-stimulating properties (10). However, the evidence for whether GC signaling promotes or inhibits tumorigenesis in nonhematologic cancer types is complex (11). Immune suppression by GCs has been associated with an increased risk of non-Hodgkin lymphomas, skin cancer, bladder cancer, and prostate cancer (12–16). In colorectal cancer, GR was reported to promote accurate chromosome segregation during mitosis, making GR a tumor-suppressor gene (17). It was also recently shown that GR deficiency in intestine leads to a hypersensitivity to acute colitis–induced colorectal cancer formation (18). Additionally, GCs have been used as an adjuvant treatment for cell cycle modulation in colorectal cancer cells (19). On the other hand, GR activation increases cancer stem cell self-renewal and chemoresistance through YAP signaling (20) and was found to promote the proliferation of metastatic colorectal cancer cells (21), suggesting an oncogenic role for GR in colorectal cancer. Therefore, our specific understanding of the role of GR in intestinal tumorigenesis remains limited.

The wide clinical use of GCs has led to an urgent need to gain a deeper understanding of GC signaling in intestinal tumorigenesis. However, the association between GR expression and colorectal cancer progression revealed in several small patient cohorts remains inconclusive. For instance, *NR3C1* (gene that encodes GR) was identified as one of the putatively crucial components of the adenomatous transformation process in 17 colorectal adenomas and paired normal mucosa (22). GR expression was also correlated with colorectal tumor histopathological characteristics and proliferative capacity, cell cycle–related molecule expression, and patient survival in 91 patients with colon cancer (19). Yet, in another study, an inverse correlation between mRNA levels of GR-α and E-cadherin was observed in colorectal adenocarcinoma (23). The long-term effects of GC use on the risk of colorectal cancer in humans are controversial. On one hand, a nested population-based cohort study by Ostenfeld et al. found that frequent use of systemic GCs was not associated with an increased overall risk of colorectal cancer in Northern Denmark (24). On the other hand, another population-based cohort study by Lai et al. reported that oral, injected, and/or topical GC use for 1–5 years before diagnosis significantly increased the risk of colorectal cancer in a Taiwanese population (25). Because of these discrepancies, understanding the causal relationship between GR signaling and chronic inflammation–associated colorectal cancer in vivo will be important for clinical management of IBD and colorectal cancer.

In the present study, we investigated the effect of GC signaling on chronic inflammation–associated tumorigenesis in humans and mice. First, using a large number of clinical samples, we observed that, compared with paired adjacent normal tissue, intestinal epithelial cells but not total tissue from colorectal cancer samples, have high GR levels, and this high epithelial GR level is associated with poor prognosis. We then generated an intestinal epithelium–specific GR-KO (GR iKO) mouse model and systematically investigated the role of intestinal epithelial GR in chemically induced colitis and chronic inflammation–induced colorectal tumorigenesis. We demonstrate that the immunosuppressive action of intestinal epithelial GR signaling ameliorated IBD but promoted chronic inflammation–induced colorectal cancer through suppression of macrophage-mediated tissue repair and antitumor responses. Therefore, our findings have major clinical implications for the use of GCs in the management of IBD and colorectal cancer. Our study also suggests that colorectal epithelial GR can serve as a predictive marker for colon cancer risk and prognosis.

## Results

### High intestinal epithelial GR expression is associated with poor prognosis in patients with colorectal cancer.

**Figure 1:**
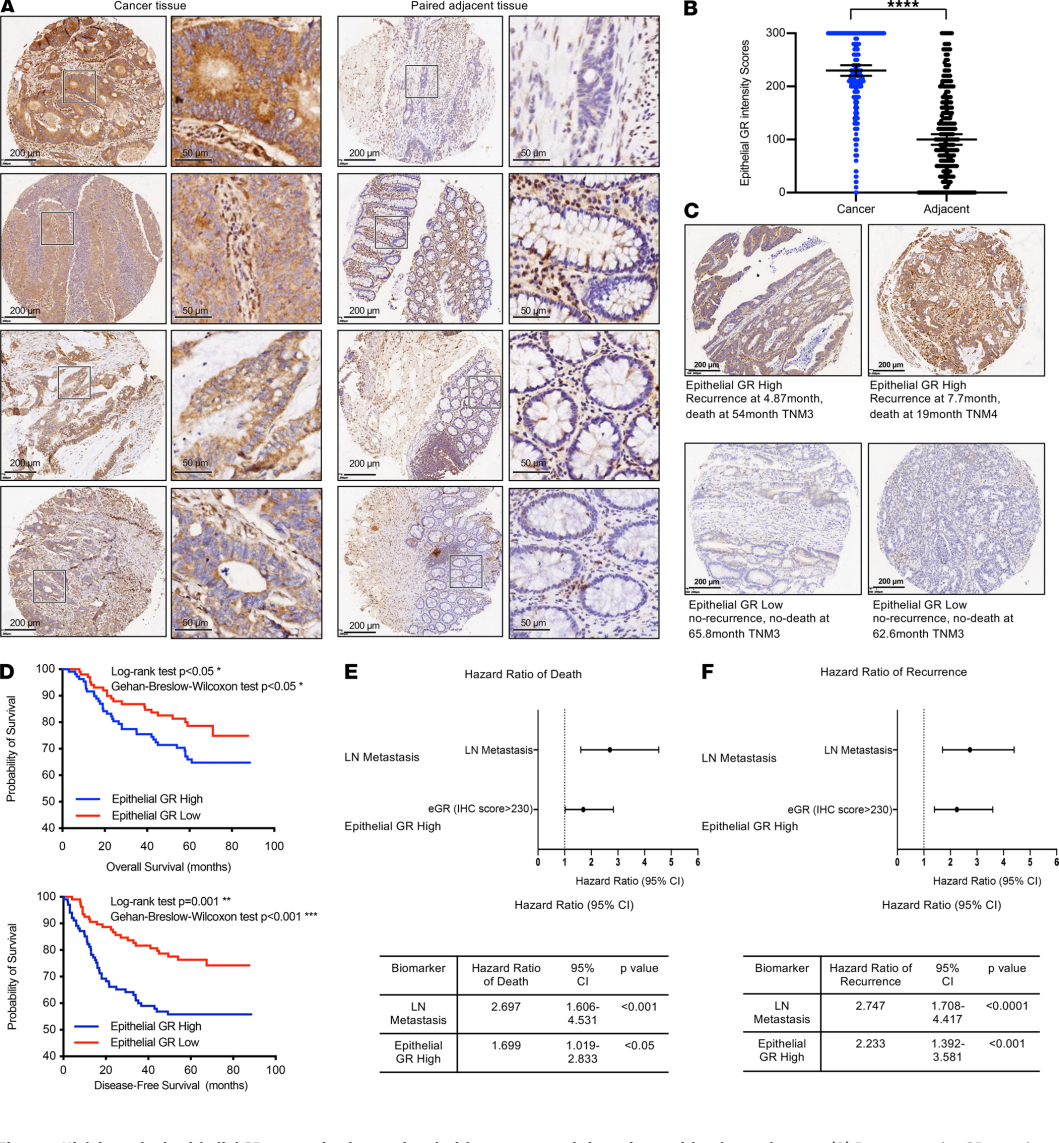
High intestinal epithelial GR expression is associated with poor prognosis in patients with colorectal cancer. (**A**) Representative GR protein IHC images of paired colorectal cancer tissue and adjacent tissues. The levels of GR protein in a colorectal cancer tissue microarray from the Fudan University Shanghai Cancer Center were determined by immunohistochemical staining with an anti-GR antibody, as described in the Methods. Scale bar: 50 μm (second and fourth column); 200 μm (first and third column). (**B**) GR is highly expressed in epithelial cells in colorectal cancer tissue compared with adjacent noncancer tissue. The GR protein staining intensity in epithelial cells was scored in 431 cancer tissues and 347 adjacent tissues, as described in the Methods (data represent mean ± SEM; **** *P* < 0.0001, Mann-Whitney test). (**C**) Representative images showing high and low epithelial GR staining in the tissue microarray and the survival data of the corresponding patients. The patients included in the microarray were stratified by dichotomizing the GR expression status in cancer tissues on a continuous H-score scale of 0–300, with a cut point of 230 (*n* = 109 for low expression and 105 for high expression). (**D**) Patients with colorectal cancer with high epithelial GR expression have significantly reduced overall survival and disease-free survival. Kaplan-Meier survival analysis was performed. (**E** and **F**) Patients with colorectal cancer with high epithelial GR expression (epithelial GR high) have significantly increased hazard ratios for death and recurrence. Patients with colorectal cancer without lymph node (LN) metastasis were used as a reference to calculate the hazard ratio of patients with LN metastasis for death or recurrence. Patients with colorectal cancer with low epithelial GR expression were used as the reference to calculate the hazard ratios for death or recurrence of the patients with high epithelial GR expression.

To better understand the impact of GC signaling on colorectal cancer development and progression, we analyzed the protein levels of GR in a colorectal cancer tissue microarray from a cohort of patients with colorectal cancer at the Fudan University Shanghai Cancer Center by immunohistochemical staining with an anti-GR antibody and scored the tissues based on the intensity and frequency of staining. The microarray consisted of 431 cancer tissues and 347 adjacent tissues from 214 patients, amounting to 1–3 tumor tissues and 1–2 paired adjacent tissues per patient. GR protein was detected in epithelial cells, immune cells, and stromal cells in cancer and adjacent tissues (Figure 1A). Intriguingly, epithelial GR protein levels but not whole-tissue GR protein levels were significantly increased in cancer tissue compared with adjacent noncancer tissue when scored on a continuous immunohistochemistry H-score scale of 0–300 (26) (Figure 1B). Using the median epithelial GR protein H-score of 230 in the cancer tissue as a cut-off, patients with cancer with high epithelial GR expression (*n* = 105, Figure 1C, top) showed significantly poorer overall survival and disease-free survival than patients with low epithelial GR expression (*n* = 109, Figure 1C, bottom, and Figure 1D). Moreover, similar to lymph node metastasis, high epithelial GR expression in patients with colorectal cancer was associated with significantly increased hazard ratios for death (Figure 1E) and recurrence (Figure 1F). Together, these results demonstrate that high epithelial GR expression is a biomarker of poor prognosis in patients with colorectal cancer.

### Intestinal epithelial GR deficiency in mice increases susceptibility to chemically induced inflammation through increased recruitment of macrophages.

**Figure 2:**
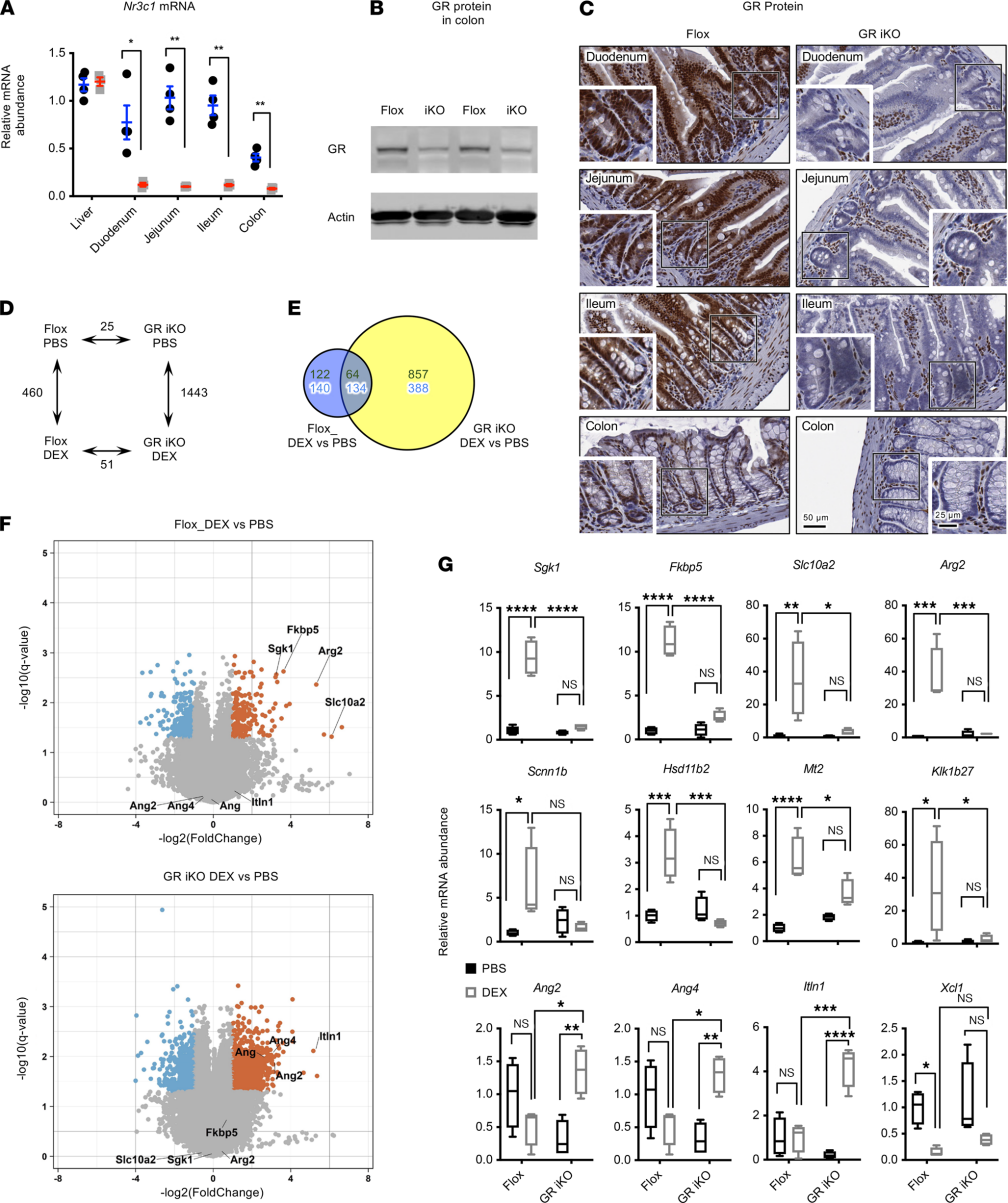
Generation of intestinal epithelium–specific GR KO mice. (**A**) mRNA levels of full-length GR in different segments of intestine and control liver tissues in Flox and intestinal epithelium–specific GR KO mice (GR iKO mice) (*n* = 4 Flox and 3 GR iKO; data represent mean ± SEM; *P < 0.05, **P < 0.01, Student’s *t* test). (**B**) GR protein is reduced in the colons of GR iKO mice. (**C**) Deletion of GR in intestinal epithelium. The protein levels of GR were analyzed by immunohistochemical staining. Insets depict depletion of GR protein in crypts of GR iKO mice. Scale bar: 50 μm; 25 μm (insets). (**D**) The numbers of significantly altered gene probes between Flox and GR iKO mice treated with PBS or DEX. ADX Flox and GR iKO mice were treated and the transcriptomes in their colons were analyzed by microarray, as described in Methods (*n* = 3 Flox and 4 GR iKO, *q* < 0.05).

To further assess the importance of intestinal epithelial GR in the regulation of intestinal tumorigenesis, we generated an intestinal epithelium–specific GR-KO (GR iKO) mouse model by crossing GR^*fl/fl*^ (Flox) mice with Villin-cre mice (as described in Supplemental Methods) (Figure 2, A–C; see complete unedited blots in the supplemental material). Under normal feeding conditions, GR iKO mice were phenotypically normal, with normal body weights, normal small intestine and colon lengths (Supplemental Figure 1, A–C; supplemental material available online with this article; https://doi.org/10.1172/jci.insight.151815DS1), and normal levels of most intestinal cell–type markers in both small intestine and colon (Supplemental Figure 1, D and E). However, microarray analysis performed after elimination of endogenous GCs by adrenalectomy (ADX) indicated that these mice displayed the expected impairment in GC signaling in response to dexamethasone (DEX), a synthetic GC (Figure 2, D–G, and Supplemental Table 1).

**Figure 3:**
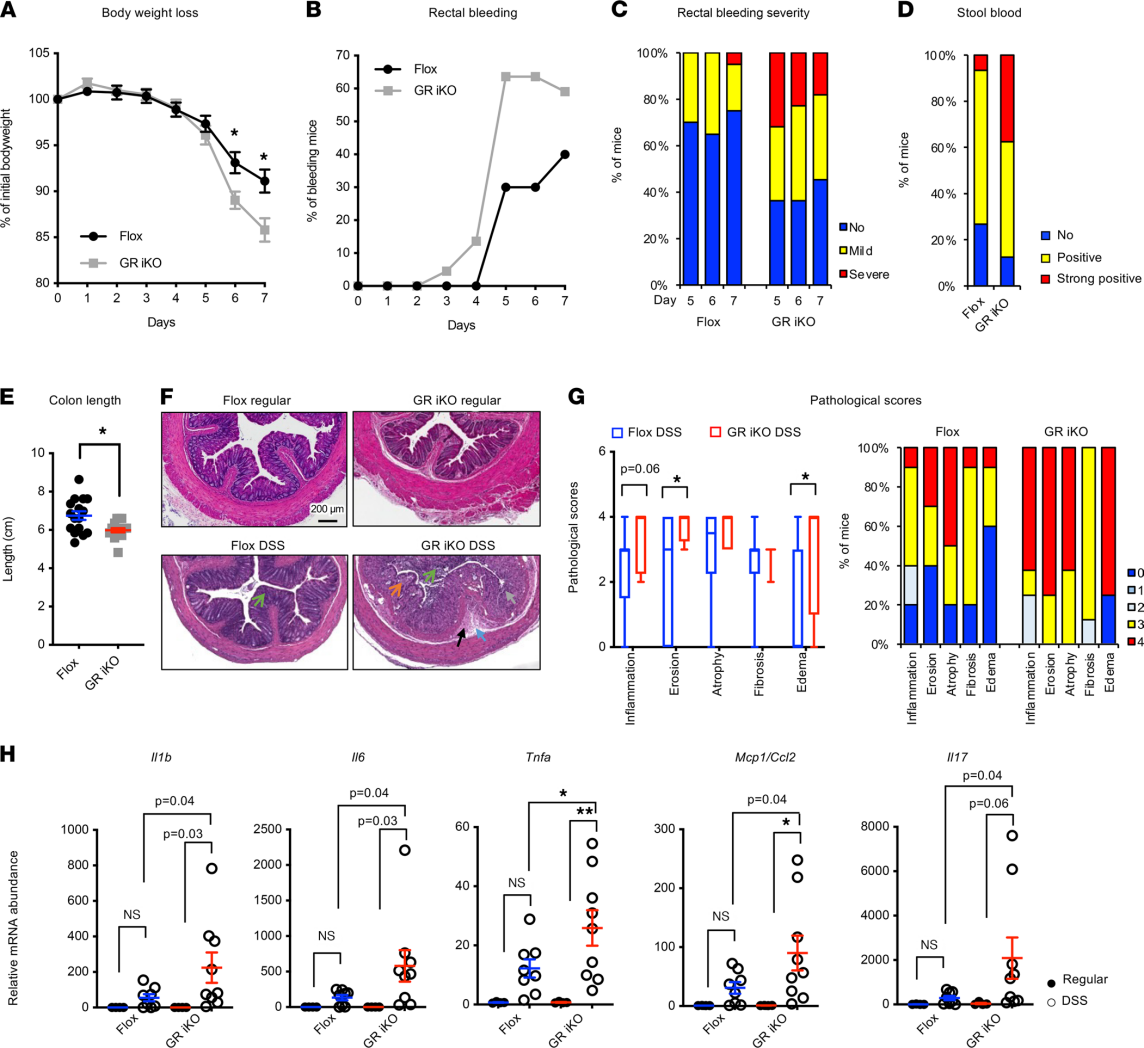
Deletion of intestinal epithelial GR increases susceptibility to DSS-induced colitis in mice. (**A–E**) Six-month-old Flox and GR iKO mice were treated with 2.5% DSS in drinking water for 7 days. (**A**) Their body weight loss, (**B**) the percentage of animals displaying rectal bleeding, (**C**) the percentages of mice by rectal bleeding severity category, (**D**) bloody stool positivity, and (**E**) the colon length were analyzed (*n* = 16 Flox mice and 17 GR iKO mice from two independent experiments). Data in (**A** and **E**) represent mean ± SEM, *P < 0.05, Mann-Whitney test. (**F** and **G**) H&E-stained colon sections from DSS-treated Flox and GR iKO mice were examined, and nonproliferative lesions were graded and recorded, as described in the Methods. Scale bar: 200 μm. The orange arrow denotes inflammation, the brown arrow denotes glandular atrophy and loss, the black arrow denotes fibrosis, the blue arrow denotes submucosal edema, and green arrows denote mucosal erosion. (**G**) Pathological scores shown with box-and-whisker plot, where whiskers represent the maximum and minimum values (*n* = 10 Flox mice and 8 GR iKO mice, *P < 0.05, Mann-Whitney test). (**H**) GR-deficient mouse colons have increased proinflammatory gene levels after DSS administration (*n* = 4 Flox mice administered regular water, 8 Flox mice treated with DSS, 4 GR iKO mice administered regular water, and 9 GR iKO mice treated with DSS; data represent mean ± SEM; *q < 0.05, **q < 0.01, 2-way ANOVA test).

GR deletion in the intestinal epithelium has recently been shown to increase the susceptibility to dextran sodium sulfate–induced (DSS-induced) colitis (18), a well-established experimental model of IBD. Consistent with this report, when challenged with 2.5% DSS in their drinking water for 7 days, our GR iKO mice experienced more body weight loss, earlier and more severe rectal bleeding, a higher frequency of and more severe bloody stool, and more extensive colonic shortening than Flox mice during the treatment (Figure 3, A–E). Accordingly, after the DSS treatment, GR iKO mice had increased colonic tissue damage, together with elevated levels of several proinflammatory cytokines/chemokines (Figure 3, F–H). Small intestines of DSS-treated GR iKO mice also displayed an increased trend in proinflammatory gene expression (Supplemental Figure 2, A–C).

**Figure 4:**
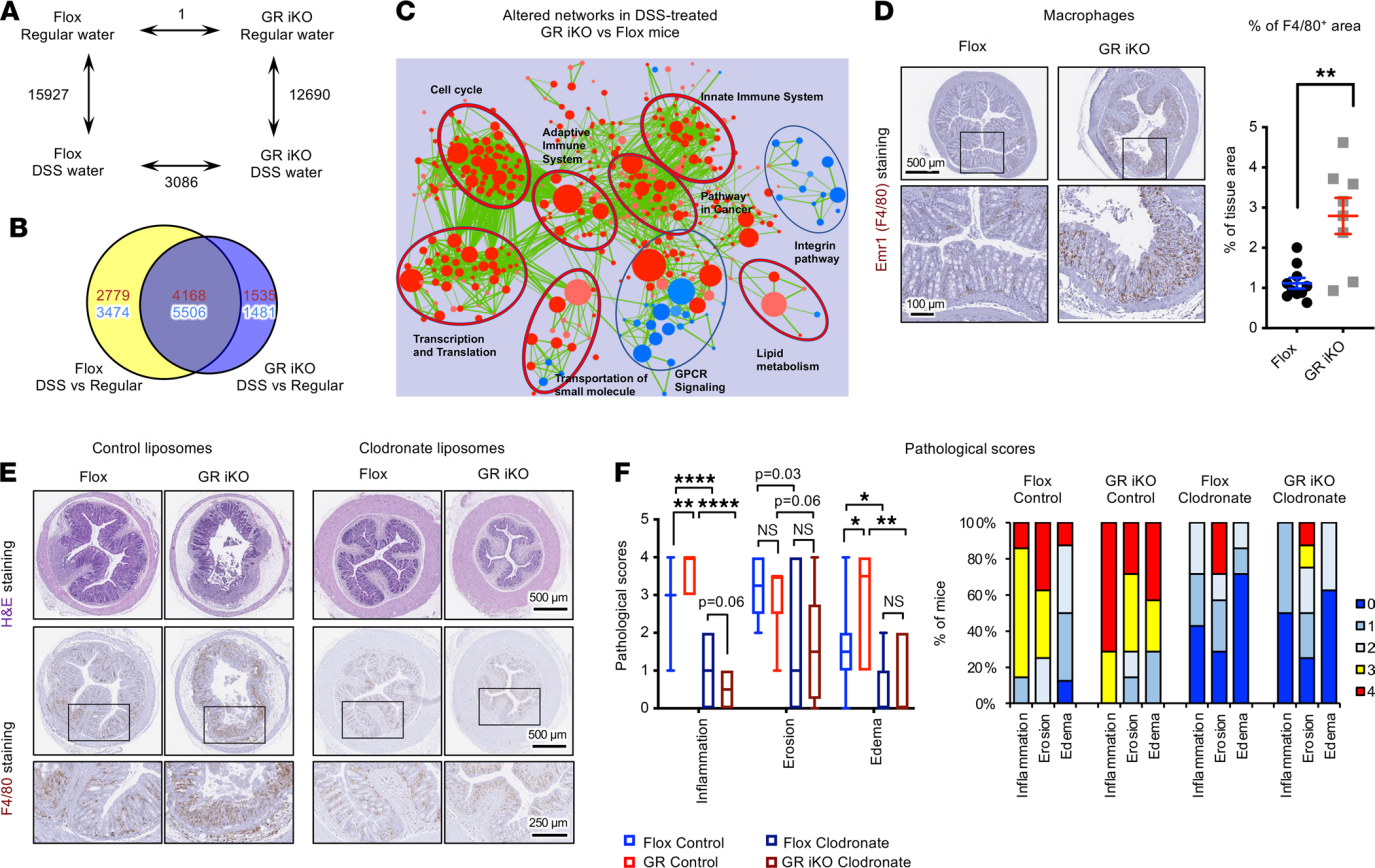
Intestinal epithelial GR deficiency enhances sensitivity to DSS-induced colitis through activation of intestinal macrophages. (**A–C**) GR iKO mice have increased immune system activation and inflammation at the transcriptomic level after DSS treatment. Six-month-old Flox and GR iKO mice were treated with regular water or with 2.5% DSS water for 7 days, and their colonic total RNA were analyzed by mouse whole genome microarray (*n* = 3–4). (**A**) The numbers of significantly altered gene probes between Flox and GR iKO mice treated with or without DSS (*n* = 3–4, *q* < 0.05). (**B**) Venn diagram representation of DSS-altered common gene probes (9674) as well as unique gene probes (6253 unique in Flox mice, and 3016 unique in GR iKO mice). Red, upregulated; blue, downregulated. (**C**) An enrichment network map of enriched gene set enrichment analysis gene sets. Red, upregulated gene sets; blue, downregulated gene sets. (**D**) DSS-treated GR-deficient mouse colons have increased infiltration of macrophages. Colon sections from DSS-treated Flox and GR iKO mice were stained with an anti-F4/80 antibody and quantified by ImageJ (NIH) (*n* = 10 Flox mice and 8 GR iKO mice; each data point represents an average of at least 3 mouse colon sections, including proximal, middle, and distal segments; data represent mean ± SEM; **P < 0.01, Mann-Whitney test). Scale bar: 100 μm (bottom); 500 μm (top). (**E** and **F**) Flox and GR iKO mice have comparable sensitivities to DSS-induced colitis after monocyte/macrophage depletion. Mice were treated as described in the Methods. Inflammation and the tissue morphology in colon H&E-stained sections were evaluated and scored as described in the Methods (*n* = 8 Flox control, *n* = 7 GR iKO control, *n* = 7 Flox clodronate, and *n* = 8 GR iKO clodronate). Scale bar: 250 μm (bottom); 500 μm (top and middle). (**F**) Pathological scores shown with box-and-whisker plot, where whiskers represent the maximum and minimum values (*q < 0.05, **q < 0.01, ****q < 0.0001, 2-way ANOVA test).

Flox and GR iKO mice also had distinct transcriptomic profiles in the colon at day 7 after DSS challenge when analyzed by microarray (Figure 4, A and B, and Supplemental Table 2). Gene set enrichment analysis showed that the colons of DSS-treated GR iKO mice had enhanced expression of gene sets involved in innate and adaptive immune networks (Figure 4C). Further immunohistochemical analysis revealed that the colons of GR-deficient mice had a greater accumulation of Emr1^+^ (also known as F4/80^+^) macrophages (Figure 4D) but not of total CD45^+^ immune cells nor CD3^+^ T cells (Supplemental Figure 2, D and E), suggesting that increased recruitment of macrophages may be accountable for the hypersensitivity of GR iKO mice to DSS-induced colitis. In support of this notion, depletion of circulating monocytes/macrophages prior to and during DSS water treatment by liposomes containing clodronate (Figure 4E, F4/80 staining) prevented tissue damage after DSS treatment (Figure 4, E and F) in both Flox and GR iKO mice. Importantly, Flox and GR iKO mice exhibited comparable degrees of DSS-induced tissue damage after treatment with clodronate-containing liposomes (Figure 4, E and F, clodronate), indicating that intestinal epithelial GR deficiency–induced hypersensitivity to DSS-induced colitis is primarily mediated by enhanced macrophage recruitment to the colonic epithelium.

GR has been previously reported to inhibit inflammation and immune activation through the repression of NF-κB (27, 28), a master regulator of inflammation; the uncontrolled activation of NF-κB has been implicated in the development of IBD (29). We found that stable knockdown of GR in CT26.WT mouse colon carcinoma cells using shRNAs (Supplemental Figure 3A; see complete unedited blots in the supplemental material) significantly enhanced the LPS-mediated induction of several NF-κB target chemokine genes but not the tested cytokine genes (Supplemental Figure 3, B and C), suggesting that the accumulation of macrophages in the colons of GR iKO mice after DSS treatment (Figure 4, D and E) may be due to increased chemokines produced by GR-deficient colonic epithelium. Collectively, we successfully generated an intestinal epithelial specific GR deficiency mouse model that is sensitive to chemically induced intestinal inflammation due to enhanced recruitment of macrophages and activation of innate immunity.

### Intestinal epithelial GR deficiency improves recovery after DSS treatment and reduces chronic inflammation–associated colorectal cancer formation.

**Figure 5:**
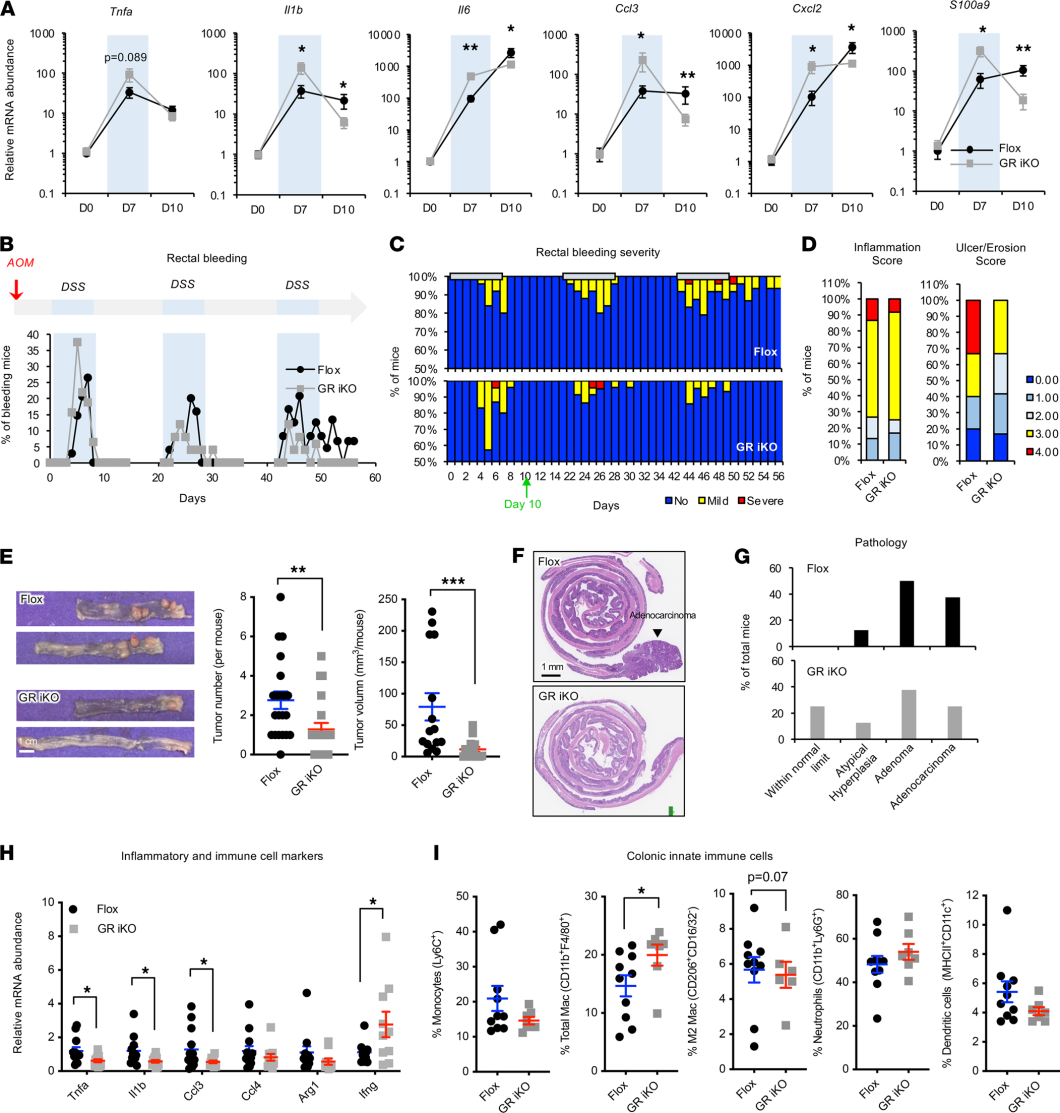
Deletion of intestinal epithelial GR enhances recovery after DSS treatment and suppresses chronic inflammation–associated colorectal cancer formation. (**A**) GR iKO mice have reduced expression of inflammatory and immune cell markers at day 10. Flox and GR iKO mice were treated with 2.5% DSS for 7 days (light blue area), followed by regular water for 3 days (D0, *n* = 4 Flox and 4 GR iKO; D7, *n* = 10 Flox and 10 GR iKO; D10: *n* = 16 Flox and 15 GR iKO). (**B** and **C**) GR iKO mice display increased improvement in rectal bleeding in an AOM/DSS colorectal cancer model. Flox and GR iKO mice were subjected to the AOM/DSS procedure, as described in Methods (*n* = 34 Flox and 32 GR iKO). (**D**) GR iKO mice show a trend of improved morphologic recovery at day 10 in the AOM/DSS model (*n* = 15 Flox and 12 GR iKO). (**E**) GR iKO mice exhibit reduced tumor formation in the AOM/DSS model (*n* = 16 Flox and 15 GR iKO). Scale bar: 1 cm. (**F**) Representative images of H&E-stained colon sections from Flox and GR iKO mice in the AOM/DSS model. Scale bar: 1 mm. (**G**) Tumor stages in Flox and GR iKO mice. The H&E-stained colon sections were evaluated and scored as described in Methods (*n* = 8 Flox and 8 GR iKO). (**H**) GR iKO mice have reduced expression of proinflammatory markers but enhanced expression of *Ifng* in the colon at the end stage of the AOM/DSS model (*n* = 11 Flox and 10 GR iKO mice). (**I**) GR iKO mice have increased infiltration of macrophages in the colon at the end stage of the AOM/DSS model, as analyzed by FACS (*n* = 20 Flox and 14 GR iKO mice; each data point represents pooled colon tissues from 2 experimental mice). Data in **A**, **E**, **H**, and **I** represent mean ± SEM; *P < 0.05, **P < 0.01, ***P < 0.001, Mann-Whitney test.

Since chronic but not acute intestinal inflammation is associated with tumor progression (6, 7), to evaluate the impact of intestinal epithelial GR on intestinal tumorigenesis in mice, we followed long-term consequences of the DSS treatment in the colons of Flox and GR iKO mice. The expression of several proinflammatory genes and *S100a9*, a prominent regulator of inflammation and myeloid cell differentiation (30, 31), was dramatically increased in the colons of both Flox mice and GR iKO mice after a 7-day treatment of DSS drinking water. Consistent with our observations that GR iKO mice were more sensitive to DSS treatment than control mice (Figures 3 and 4), the induction of these genes was significantly elevated in GR iKO mice compared with that in Flox mice (Figure 5A, day 7). After 3 days of recovery with regular water, reduced levels of *Il1b, Tnfa, Ccl3*, and *S100a9* were observed in Flox mice (Figure 5A, day 10). However, surprisingly, GR iKO mice showed even greater downregulation of these genes and attenuated induction of *Il6* and *Cxcl2* after the 3-day recovery period (Figure 5A, day 10), suggesting that immune hyperactivation upon DSS treatment in GR iKO mice is associated with improved recovery after the treatment.

To further assess the unexpected impact of intestinal epithelial GR deficiency on tissue recovery, we directly challenged Flox and GR iKO mice with an azoxymethane/DSS (AOM/DSS) chronic inflammation–associated colorectal cancer model, in which the mice were first injected with AOM to induce DNA damage and then treated with 3 cycles of alternating DSS-containing water and regular drinking water to induce chronic inflammation and repetitive tissue damage/repair (Figure 5B, top). Although a proportion of GR iKO mice experienced more severe rectal bleeding than Flox mice in the first DSS treatment cycle, a reduced fraction of GR iKO mice relative to Flox mice experienced rectal bleeding during the later DSS cycles (Figure 5B, bottom, and Figure 5C). Histological evaluation of colon tissues dissected after 3 days of regular water recovery in the first treatment cycle (day 10) further revealed that GR iKO mice exhibited a trend of less severe tissue damage (Figure 5D), diminished tissue macrophage accumulation, and increased epithelial proliferation (Supplemental Figure 4, A and B) compared with Flox mice. These observations indicate that the hyperactivation of the immune system in the GR-deficient colon upon DSS challenge, though it enhances susceptibility during the acute colitis stage, is associated with improved tissue recovery and healing after the DSS treatment.

Consistent with their improved recovery after the DSS treatment cycles, GR iKO mice developed fewer and smaller colorectal tumors than Flox mice at the end of the chronic AOM/DSS experiment (Figure 5E). Histopathological analyses further revealed that all analyzed Flox mice developed hyperplasia, adenoma, and/or adenocarcinoma, with more than 85% of them bore tumors that were at advanced adenoma or adenocarcinoma stages (Figure 5, F and G, Flox). In contrast, 25% of GR iKO mice did not have any tumors (Figure 5, F and G, GR iKO). Additional IHC staining analysis showed that colorectal tumors developed in GR iKO mice had a comparable intensity of Ki67 staining but a trend of increased TUNEL staining compared with those from Flox mice (Supplemental Figure 4, C and D). These observations indicate that GR iKO mice have a lower risk of developing advanced colorectal cancer than Flox mice.

Intriguingly, at the end of the chronic AOM/DSS experiment, GR iKO mice displayed a lower trend of inflammatory histology in the colon compared with Flox mice (Supplemental Figure 4E), along with markedly lower mRNA levels of most tested proinflammatory genes (Figure 5H). However, the expression of *Ifng*, which encodes IFN-γ (a key cytokine with multiple antitumor activities that is particularly important in stimulating the tumoricidal activity of macrophages; ref. 32), was significantly increased in the colons of GR iKO mice compared with those of Flox mice (Figure 5H). This observation raises the possibility that the observed reduction in colorectal tumorigenesis in GR iKO mice may be associated with an enhanced macrophage-mediated antitumorigenic response. Indeed, FACS analyses showed that, at the endpoint of the AOM/DSS experiment, the GR iKO mice exhibited increased infiltration of macrophages in the colon compared with that in Flox mice (Figure 5I and Supplemental Figure 4F). In contrast, the tissue abundance of several other analyzed immune cell types was comparable between Flox and GR iKO mice (Figure 5I and Supplemental Figure 4G). Taken together, our results suggest that intestinal epithelial GR deficiency induces a macrophage-mediated antitumorigenic response, which in turn helps improve tissue repair and recovery after DSS treatment and eventually reduces chronic inflammation–associated colorectal cancer formation.

### Early-phase GC treatment impairs recovery from colitis and promotes chronic inflammation–associated colorectal cancer.

**Figure 6:**
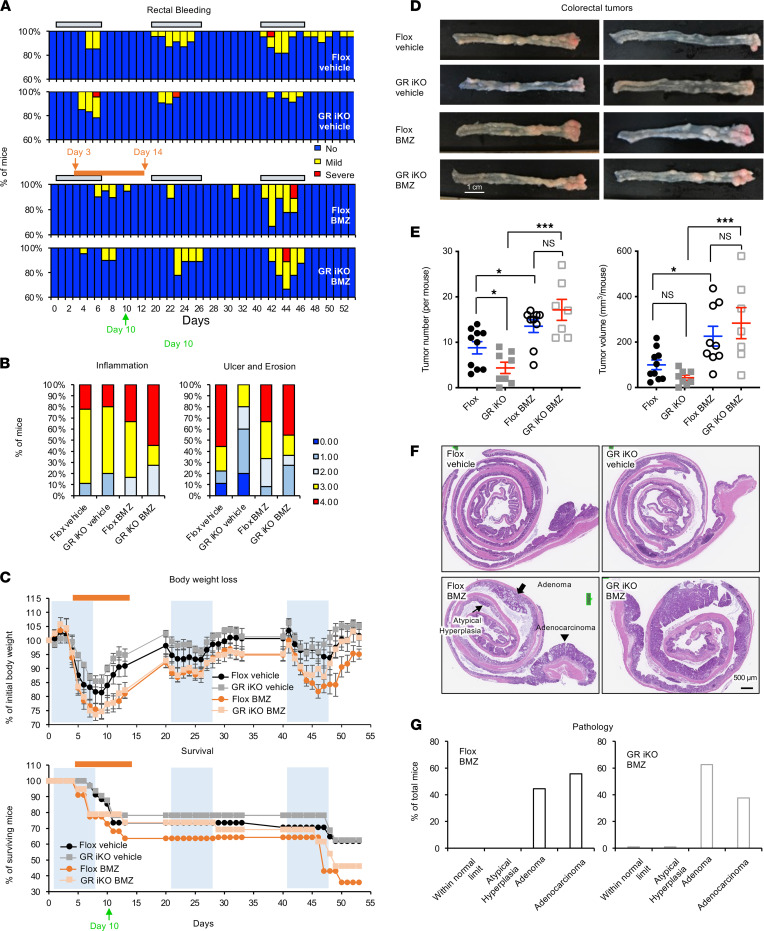
Early-phase betamethasone treatment increases AOM/DSS-induced colorectal cancer formation. (**A–C**) Eleven-day oral betamethasone (BMZ) treatment during the first DSS treatment/recovery cycle impairs recovery of rectal bleeding of both Flox and GR iKO mice in an AOM/DSS colorectal cancer model. Three- to four-month-old Flox and GR iKO mice under AOM/DSS treatment were subjected to oral treatment of either vehicle (0.125% ethanol) or 2.5 μg/ml BMZ from day 3 to day 14. (**A**) The rectal bleeding severity during the entire experimental time frame (initial animal numbers, *n* = 28 Flox vehicle, 27 GR iKO vehicle, 21 Flox BMZ, and 22 GR iKO BMZ). (**B**) Their histological scores of inflammation (left) and morphology (right) were evaluated by a professional pathologist at day 3 during the first regular water recovery phase (day 10) (*n* = 15 Flox vehicle, 12 GR iKO vehicle, 12 Flox BMZ, and 11 GR iKO BMZ). (**C**) The body weight loss and surviving animals (initial animal numbers, *n* = 28 Flox vehicle, 27 GR iKO vehicle, 21 Flox BMZ, and 22 GR iKO BMZ). (**D** and **E**) Oral BMZ treatment during the first DSS treatment/recovery cycle increases colon tumor formation in both Flox and GR iKO mice in an AOM/DSS colorectal cancer model (*n* = 10 Flox vehicle, 8 GR iKO vehicle, 9 Flox BMZ, and 7 GR iKO BMZ; data represent mean ± SEM; *q < 0.05, ***q < 0.001, 2-way ANOVA test). (**F** and **G**) Colorectal cancers developed in BMZ-treated mice are at more advanced stages compared with vehicle-treated mice in an AOM/DSS colorectal cancer model. The tumor histology and stages were evaluated by a professional pathologist, as described in Supplemental Methods (*n* = 9 Flox BMZ, and *n* = 7 GR iKO BMZ).

GCs are one of the most clinically prescribed medicines for inflammation inhibition and immunosuppression, including treatment of IBD (5). However, our observations that GR iKO mice suffered more severe acute colitis than Flox mice yet were partially protected from chronic inflammation–induced colon tumorigenesis suggest that early-phase immunosuppression may actually delay recovery from intestinal inflammation/damage and promote chronic inflammation–associated cancer formation. To directly test this possibility, we treated Flox and GR iKO mice subjected to the AOM/DSS procedure with drinking water containing 2.5 μg/ml betamethasone (BMZ), a synthetic GC, from day 3 in the first DSS cycle (day 3) to 7 days into the first recovery phase (day 14) (Figure 6A, orange bar) and analyzed the effect of this treatment on DSS-induced inflammation and colorectal cancer formation at the end of the AOM/DSS procedure. This BMZ treatment time frame was chosen based on our observation that day 3 to day 7 in the first DSS cycle was the major period of tissue damage in the GR iKO mice (Figure 5C). As shown in Figure 6A, early-phase BMZ treatment reduced rectal bleeding in both Flox and GR iKO mice during the first DSS cycle. However, continued rectal bleeding was observed in a fraction of BMZ-treated mice during the first recovery phase (Figure 6A, BMZ), and histological scores for inflammation and ulceration/erosion on day 10 were increased in BMZ-treated mice compared with vehicle-treated mice (Figure 6B), indicating delayed healing in the BMZ-treated mice during the first recovery phase. Consequently, early-phase BMZ treatment led to increased body weight loss and reduced survival (Figure 6C and Supplemental Figure 5A) and more severe rectal bleeding in the last DSS cycle among the surviving mice (Figure 6A). The observed comparable responses of Flox and GR iKO mice to this early-phase GC treatment further indicate that BMZ-mediated intestinal immunosuppression is independent of intestinal epithelial GR, possibly through inhibition of immune cell activation. In agreement with this idea, BMZ repressed the expression of *Ifng* and *Nos2*, an inducible nitric oxide synthase primarily expressed by immune cells, including macrophages (33), in the colons of both Flox ang GR iKO mice immediately after the BMZ treatment at day 14 (Supplemental Figure 5B).

Consistent with the above observations, early-phase BMZ–treated mice developed more and larger colorectal tumors than vehicle-treated mice (Figure 6, D and E), and these adenoma or adenocarcinoma tumors were at a more advanced stage (Figure 6, F and G). The complete reverse of the reduced colorectal tumor progression in GR iKO mice by BMZ is in line with the notion that intestinal epithelial GR deficiency decreases intestinal tumor growth by increasing immune cell activation and function.

**Figure 7:**
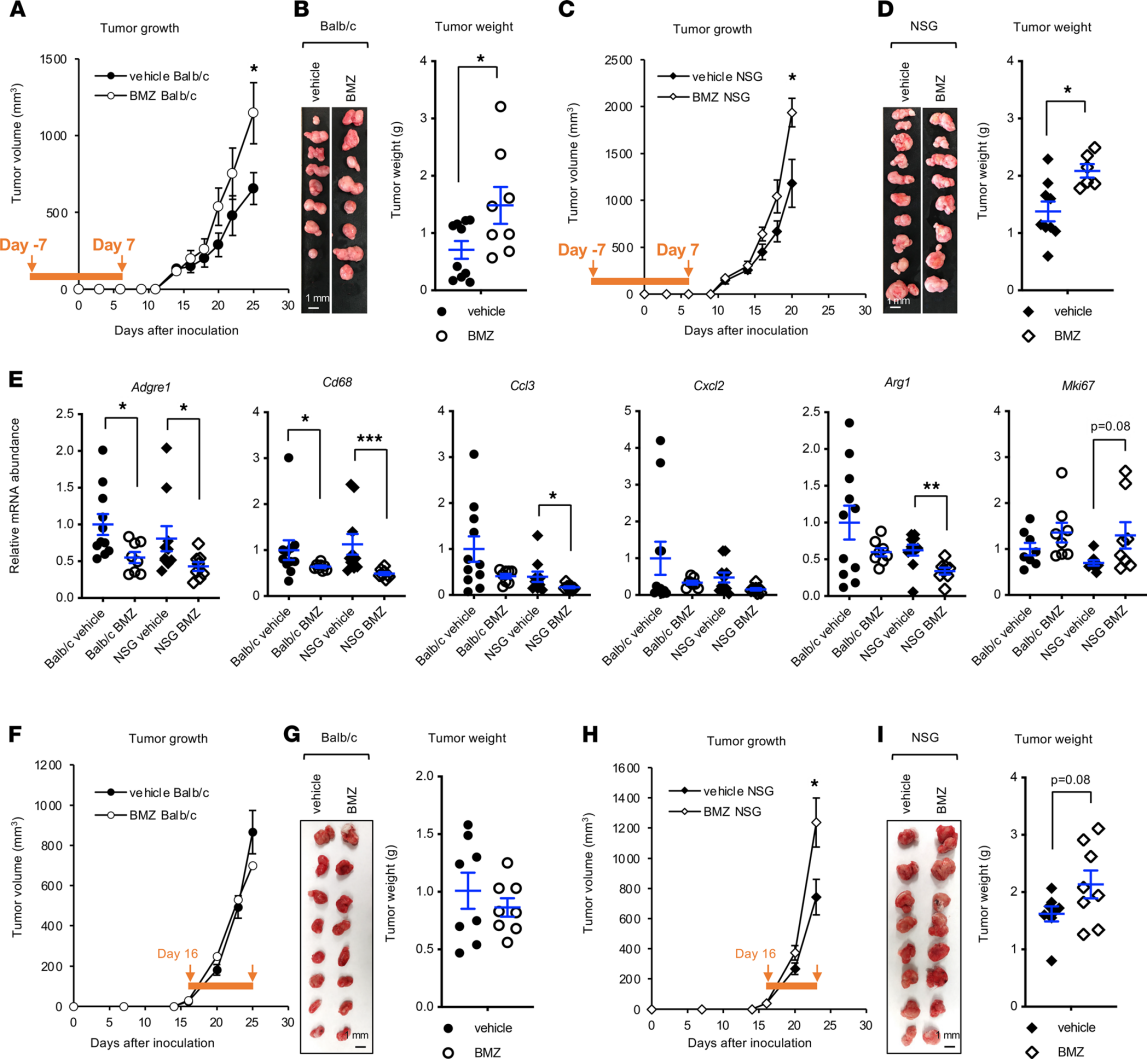
Early-phase betamethasone treatment increases the growth of allografted colon carcinoma cells in mice. (**A** and **B**) Early-phase oral BMZ treatment increases the growth of allografted CT26.WT carcinoma cells in BALB/c mice. Two-month-old immunocompetent BALB/c mice were treated and analyzed as described in the Methods. (**A**) Tumor volume was monitored, and (**B**) a final image of the dissected tumors as well as tumor weights are shown (*n* = 8–10; data represent mean ± SEM; *P < 0.05, Mann-Whitney test). (**C** and **D**) Early-phase oral BMZ treatment increases the growth of allografted CT26.WT colon carcinoma cells in NSG mice (*n* = 8–10, one outlier from each group was removed for tumor weight analysis using IQR analysis, in which any values outside the 1.5 × IQR range were considered outliers; data represent mean ± SEM; *P < 0.05, Mann-Whitney test). (**E**) The expression of macrophage markers, chemokines, and proliferation genes in allografted CT26.WT colon tumors. The allografted CT26.WT colon tumors in **B** and **D** were analyzed for the expression of the indicated genes by qPCR (*n* = 8–10; data represent mean ± SEM; *P < 0.05, **P < 0.01, ***P < 0.001, Mann-Whitney test). (**F–I**) Late-phase oral BMZ treatment increases the growth of allografted CT26.WT colon carcinoma cells in NSG mice but not BALB/c mice. Two-month-old immunocompetent BALB/c mice and NSG mice were allografted with CT26.WT cells, and tumor volume was monitored. When the average tumor size reached 30–40 mm^3^ (at day 16), mice in each strain were randomly divided into 2 groups and treated with vehicle or BMZ for the rest of the experiment (*n* = 8 tumors/group for both BALB/c mice and NSG mice; data represent mean ± SEM; *P < 0.05, Mann-Whitney test).

To further confirm that early-phase GC treatment could indeed enhance tumor progression at the late stage through immunosuppression, we employed a syngeneic tumor model using CT26.WT mouse colon carcinoma cells that can form tumors in immunocompetent BALB/c mice (34). We pretreated immunocompetent BALB/c mice with either vehicle or 2.5 μg/ml BMZ for 7 days, s.c. allografted CT26.WT cells, and then continued the same treatment protocol for an additional 7 days (Figure 7A, orange bar, day –7 to day 7). Notably, this BMZ treatment increased late-stage CT26.WT tumor volume (Figure 7A, day 26) and final tumor weight (Figure 7B). To test whether suppression of macrophage infiltration is involved in the BMZ-mediated increase of CT26.WT tumor growth, we repeated the BMZ-allograft procedure in NOD/SCID γ (NSG) mice, one of the most immunocompromised mouse strains that lacks mature T, B, and natural killer cells due to the *scid* mutation and *Il2rg* KO (35) but with functional macrophages (36, 37). NSG or SCID mice have been used to assess macrophage-specific functions in human red blood cell reconstitution, cancer rejection, and obesity (36–39). Intriguingly, the same BMZ treatment scheme also increased late-stage CT26.WT tumor volume (Figure 7C) and weight (Figure 7D) in NSG mice, suggesting that early-phase BMZ treatment enhances tumor growth through suppressing macrophages. Indeed, BMZ reduced the mRNA levels of *Adgre1* (also known as *F4/80*) and *Cd68*, two macrophage markers, and several chemokine genes in dissected allografted tumors (Figure 7E).

To further test whether there is a therapeutic window for GC therapy during colon cancer development, we initiated the BMZ treatment at a late stage with visible allograft CT26.WT tumors (day 16) in the syngeneic tumor model (Figure 7, F and G). In contrast to the early-phase BMZ administration, this late-phase BMZ treatment did not significantly promote tumor growth in BALB/c mice (Figure 7, F and G), indicating that GC treatment will not induce further complications once tumors are established in immunocompetent animals. However, interestingly, the same late-phase BMZ treatment was still able to promote tumor growth in NSG mice (Figure 7, H and I), suggesting that GC therapy is detrimental for immunocompromised individuals at any stages. In summary, our results demonstrate that early-phase systemic GC treatment, while effective in suppressing acute inflammation, impairs subsequent recovery from acute colitis and thereby promotes chronic inflammation–associated colorectal cancer progression, at least in part through suppressing macrophages.

## Discussion

As a class of complex inflammatory disorders affected by various genetic and environmental factors, IBDs lack a specific treatment. Instead, current treatments focus on alleviating inflammation, and GCs serve as a first-line therapy for patients with modest-to-severe symptoms (5). But the relationship of GR with colorectal cancer in clinic samples is inconclusive. Here, we provide evidence that intestinal epithelial GR signaling reduces intestinal inflammation upon acute chemically induced damage. However, this response delays tissue repair, increasing colorectal cancer formation at a later phase (as depicted in Supplemental Figure 6). Our study therefore indicates that intestinal epithelial GR signaling, activated by endogenous or exogenous GCs, promotes chronic inflammation–associated colorectal cancer formation.

Emerging evidence indicates that the increase in stress hormones during cancer progression is able to activate GR in cancer cells and promote tumor formation. In breast cancer, this activation increases tumor heterogeneity and metastasis through multiple processes, including the kinase ROR1 (40). Psychological distress and/or cancer-induced negative mood in colorectal cancer and non–small cell lung carcinoma have also been shown to elevate plasma corticosterone levels and induce Tsc22d3 in dendritic cells, blocking type I interferon responses and dampening therapy-induced anticancer immunosurveillance (41). Our observations in the present study demonstrate that high intestinal epithelial GR expression is associated with poor prognosis in patients with colorectal cancer, and conversely, intestinal epithelial-specific GR deficiency promotes tissue repair and reduces the development of advanced colorectal cancer in mice. Our findings further suggest that activation of endogenous GR signaling in colorectal cancer cells can drive tumor progression through suppression of macrophage recruitment and subsequent tissue healing. Thus, activation of GR signaling by environmental or emotional stress not only directly promotes heterogeneity/metastasis in tumor cells and blocks anticancer activity in immune cells, but also disrupts the communication between epithelial and immune cells, which in turn enhances evasion of damaged cells from immune surveillance and accelerates tumor progression.

Several lines of evidence from the literature support the hypothesis that using GC during the acute inflammatory phase after surgically induced colon tissue damage is detrimental for injury repair and tissue healing during colorectal cancer development. For instance, in a colorectal cancer cohort evaluated by Ostenfeld et al., frequent use of systemic GCs was not associated with an increased overall risk of colorectal cancer (24); instead preadmission use of oral GCs was associated with increased 30-day mortality (42) and a greater risk of anastomotic leakage (43) after rectal cancer resection. Moreover, preoperative DEX therapy was shown to be associated with an elevated rate of distant recurrence in patients undergoing colectomy for colorectal cancer (44). These reports are consistent with our finding that early-phase oral GC administration delayed recovery after colitis and increased the development of colorectal cancer at a later stage.

Notably, our study is agreement with a recent study by Muzzi et al., in that deletion of intestinal epithelial GR in mice aggravates DSS-induced acute colitis (18). However, the study by Muzzi et al. reported that GR iKO mice are hypersensitive to acute colitis–induced colorectal cancer formation in an acute AOM/DSS colorectal cancer model (18), which is in contrast with our observation that GR iKO mice are protected from colorectal cancer formation in a chronic inflammation–associated AOM/DSS colorectal cancer model. Careful comparison revealed that the GR iKO mouse models used in these two studies are generated differentially. Our GR iKO mouse model is an intestinal epithelium–specific constitutive KO line (driven by Villin-cre), which does not require any additional manipulations prior to experimental procedures. Moreover, we included both male and female animals in our study. The GR iKO line in the Muzzi et al., in contrast, is a tamoxifen-inducible mouse strain (driven by Villin-cre-ERT). They also only used female mice in their study. Tamoxifen is a selective modulator of estrogen receptor (45, 46), which is known to interact with GC signaling (47, 48). Moreover, tamoxifen has been shown to repress leukocyte infiltration (49) and increase the risk of large bowel cancer in females (50). Therefore, it is highly likely that the tamoxifen dosing prior to experimental procedures interfered with their experimental outcomes (including reduction of leukocyte infiltration and increase of tumor formation). Another possible contributing factor to this discrepancy may be the different types of inflammation associated colorectal cancer models in the two studies. The AOM/DSS colorectal cancer model employed by Muzzi et al. involved 1 cycle of 1.2% DSS treatment with AOM injection. As chronic intestinal inflammation is an important risk factor for the development of colorectal cancer, we chose an AOM/DSS colorectal cancer model involving 3 cycles of 2.5% DSS water. Therefore, it is likely that the single cycle of 1.2% DSS in the Muzzi et al. study did not mimic chronic inflammation in the intestine and that the enhanced colorectal cancer formation in the Muzzi et al. model reflects the effect of intestinal epithelial GR signaling on acute inflammation. In contrast, treatment with 3 cycles of 2.5% DSS in regular drinking water, the protocol used in our study, induced chronic inflammation and repetitive tissue damage/repair, which mainly engaged the action of intestinal epithelial GR signaling to suppress tissue healing. Future studies are needed to validate these possibilities.

Our study has a number of important clinical implications. First, the effect of early-phase GC treatment on colorectal cancer development uncovered in our study has implications in IBD treatment in the clinic, which suggests that GC treatment for IBD should be used with caution. Second, our clinical findings in patients with colorectal cancer indicate that colorectal epithelial GR, but not total colonic tissue GR, could serve as a predictive marker for colorectal cancer risk and prognosis. Third, our observations that early-phase GC treatment is detrimental for late-phase tumor growth and that late-phase GC treatment did not significantly affect tumor growth in immunocompetent animals suggest that there is a therapeutic window for GC therapy during colorectal cancer development in immunocompetent patients. At the precancer or early inflammation-associated initiation stage, GC treatment would prevent a much-needed immune response that would contain damaged and mutated cells and, therefore, is oncogenic. Once tumors are established, GCs may be helpful for managing the side effects of standard cancer therapies without further complications. Finally, the chronic inflammation–associated colorectal cancer model employed in our study revealed an important role of the immunosuppressive action of GR in promoting colorectal cancer progression. This finding is supported by our clinical observation that high intestinal epithelial GR expression is associated with poor prognosis in patients with colorectal cancer and is also consistent with the report that psychological distress and/or cancer-induced negative mood–associated elevation of plasma corticosterone levels reduce anticancer immunotherapy in colorectal cancer (41). Our chronic inflammation–associated colorectal cancer model is therefore a clinically relevant colorectal cancer model.

In summary, our study has provided solid evidence that intestinal epithelial GR promotes chronic inflammation–induced colorectal cancer and has revealed an unexpected relationship between early-phase oral GC administration and late-phase colorectal cancer development. Our findings suggest that GC therapy for IBD should be applied with caution and that colonic epithelial GR could serve as a predictive marker for colorectal cancer. Our findings further suggest that identification of an appropriate GC treatment window is needed for future GC usage in IBD and colorectal cancer. Our study therefore has important translational implications for the management of these human diseases.

## Methods

Further information can be found in Supplemental Methods.

### Human studies

GR protein levels in a tissue microarray established by the Fudan University Shanghai Cancer Center were analyzed by immunohistochemistry with an anti-GR antibody (catalog 3660, Cell Signaling Technology). This microarray consists of 431 cancer tissues and 347 adjacent tissues from 214 patients with colorectal cancer with follow-up data, including 127 Asian men and 87 Asian women, with an average age of 57.2 ± 10.9 (mean ± SD) (Table 1). The histological staining results were scored on a continuous immunohistochemistry H-score scale of 0–300 by the integration of data relating to the intensity and frequency of staining, and the immunohistochemistry score was calculated with the formula: 1 × (percentage of cells staining weakly [1+]) + 2 × (percentage of cells staining moderately [2+]) + 3 × (percentage of cells staining strongly [3+]), as previously published (26). The cutoff point to define high and low epithelial GR expression in the colorectal cancer tissues was the median IHC score for all the colorectal cancer tissues. Both the log-rank test and Gehan-Breslow-Wilcoxon test were performed to calculate the significance of differences in survival. The Mantel-Haenszel χ^2^ test was used to calculate the hazard ratios for death and recurrence.

### Animal experiments

To test the transcriptional responses of Flox and GR iKO mice to DEX, 3-month-old male Flox and GR iKO mice underwent ADX. After 2 weeks of rest, the mice were injected i.p. with 1 mg/kg body weight DEX in PBS (100 μl), and tissues were harvested after 6 hours.

Acute colitis was induced in mice using 2.5% DSS in the drinking water with two different protocols: (a) treat mice with DSS water for 7 consecutive days; (b) treat mice with DSS water for 7 days and then replace DSS water with regular drinking water to allow recovery for up to another week. The body weights of the experimental mice and rectal bleeding were monitored daily, and stool blood scores were measured at the end of the treatment period. Rectal bleeding severity was scored using a previously described scale (51) (0, none; 1, red; 2, dark red; and 3, gross bleeding) and was graded as none (score 0), mild (score 1), or severe (scores 2 and 3).

To test the importance of macrophages in mediating intestinal epithelial GR deficiency–induced sensitivity to DSS-induced colitis, Flox and GR iKO mice were i.p. injected with control liposomes or liposomes containing clodronate at a dose of 10 mg/kg body weight 1 day prior to administration of 2.5% DSS in the drinking water and then once daily during DSS treatment to deplete circulating monocytes/macrophages and reduce their infiltration into the intestinal epithelium. The depletion of monocytes/macrophages was monitored in blood at day 5 after treatment by FACS (data not shown).

For the AOM/DSS colorectal cancer model, 3- to 4-month-old mice were injected with AOM (8 mg/kg body weight). One week later, the mice were fed 2.5% DSS-containing water for 6–7 days, followed by a 14-day recovery period with regular drinking water; this treatment cycle was repeated 3 times. Rectal bleeding severity was scored and graded as described above for the DSS-induced colitis model. Intestinal and colonic tissue were harvested 10–12 weeks after DSS treatment. For early-phase BMZ treatment in this model, 3- to 4-month-old control mice under AOM/DSS treatment were subjected to oral treatment with either vehicle (0.125% ethanol) or 2.5 μg/ml BMZ in 0.125% ethanol in drinking water from day 3 to day 14, and body weight, rectal bleeding severity, and survival were monitored during the entire experimental time frame.

Two protocols were used for allograft tumor experiments using mouse colon carcinoma cell line CT26.WT (ATCC, CRL-2638). In the first protocol, 2-month-old female BALB/c mice (000651, The Jackson Laboratory) and female NSG mice (005557, The Jackson Laboratory) were pretreated with either vehicle (0.125% ethanol) or 2.5 μg/ml BMZ in the drinking water for 7 days, and then 1 × 10^5^ CT26.WT cells were injected s.c. into each flank. The same treatment then continued for an additional 7 days, before it was switched to regular water. In the second protocol, BALB/c and NSG mice were injected with 1 × 10^5^ CT26.WT cells first, and the tumor growth was then monitored twice weekly. When tumors became palpable (average tumor size reached 30–40 mm^3^ at day 16), BALB/c and NSG mice were randomly divided into 2 groups and treated with either vehicle (0.125% ethanol) or 2.5 μg/ml BMZ in the drinking water for the rest of the experiment. The experimental mice in both protocols were monitored twice weekly for tumor growth and overall health. Tumor length and width were measured with calipers, and tumor volume was calculated using the formula *V* = length × width^2^/2. Mice were sacrificed when their total tumor volume reached 2000 mm^3^, according to the approved animal protocol.

### Histopathologic evaluation of DSS-induced colitis tissue samples

To evaluate the histopathology of Flox and GR iKO mice treated with DSS-containing drinking water, H&E-stained sections of the large intestine (colon) were examined, and nonproliferative lesions were graded and recorded by a professional pathologist based on a previously described 4-point scale (Table 2) (52); they were additionally described and recorded based on standard diagnostic criteria (53). Specifically, inflammation, chronic active accompanied mucosal erosion, was characterized by the intense infiltration of neutrophils with relatively few lymphomononuclear cells. Erosion was defined as significant loss of intestinal glandular epithelial cells (enteroendocrine and surface absorptive cells) and goblet cells, resulting in effacement of the mucosal architecture. Glandular atrophy (mucosal atrophy) was characterized by the presence of shortened or absent intestinal glands (crypts of Lieberkuhn) in the lamina propria. Fibrosis was characterized by the presence of proliferating fibroblasts with enlarged, oval nuclei, with extended cytoplasmic processes within the lamina propria. Edema was characterized by the accumulation of an excessive amount of fluid resulting in expansion of the submucosa.

### Evaluation of AOM/DSS colorectal cancer tissue samples

Tumors formed in the colons of AOM/DSS-treated mice were counted. The length and width of each tumor were measured with calipers, and tumor volume was calculated using the formula volume = length × width^2^/2. The total tumor volume in each mouse was calculated by adding the volumes of all tumors formed in that mouse.

To evaluate the histopathology of AOM/DSS-treated mouse colons, H&E-stained Swiss roll sections of the large intestine (colon) were blindly evaluated by a professional pathologist for the presence of tumors, staging of tumors, and presence of proliferative and nonproliferative lesions (inflammation and ulceration, respectively). The following lesion descriptions, morphologic diagnoses, and severities were used:

#### Atypical hyperplasia.

Atypical hyperplasia was characterized by crypts lined by crowded epithelial cells that maintained polarity and some dysplasia. This type of lesion was smaller in size and less proliferative than would be expected for an adenoma or adenocarcinoma.

#### Adenoma.

Adenomas were composed of either proliferations of branching tubules in the lamina propria or finger-like projections of lamina propria lined by proliferating epithelium.

#### Adenocarcinoma.

Adenocarcinoma was defined as a sessile proliferation of epithelial cells, with invasion into the underlying lamina propria and submucosa.

#### Acute inflammation.

Acute inflammation was characterized as the infiltration — predominantly of neutrophils, with fewer lymphocytes, plasma cells, and macrophages — within the lamina propria and submucosa or even extending through the muscularis to the serosa, depending on the severity. Edema could be observed in the submucosa; the degree of edema roughly paralleled the inflammation severity. Severity grading was performed subjectively based on the number of inflammatory cells, the extent of the inflammation, and other associated changes, such as edema in the submucosa. With minimal acute inflammation, there were scattered neutrophils within the lamina propria (+1); mild inflammation (+2) was recorded when there were more neutrophils in the lamina propria and infiltration into the submucosa; submucosal edema was also sometimes present to a mild degree. Moderate (+3) and marked inflammation (+4) were characterized by progressive increases in the number of inflammatory cells; involvement of the submucosa, muscularis, and serosa; associated submucosal edema; and the accumulation of inflammatory cells and cell debris in the lumen of the colon.

#### Ulcer.

Ulcers in the large intestine were characterized by a complete loss of the surface epithelium in the tunica mucosa down to the level of the submucosa. Ulcer severity scores were based on the amount of mucosa that was ulcerated, with a minimal score (+1) representing ulceration of approximately 25% or less of the surface epithelium; mild ulceration (+2) involving approximately 26%–50% of the surface epithelium; moderate ulceration (+3) indicating ulceration of approximately 50%–75% of the surface epithelium; and marked ulceration (+4) involving greater than 75% of the mucosa in the section evaluated.

#### Erosion.

Erosion of the epithelium of the anus was characterized by necrosis of the epithelium that did not extend below the basement membrane of the epithelium; that is, the necrosis did not extend into the lamina propria.

### Isolation of immune cells from the colon

CD45^+^ immune cells were isolated from the colon tissues of Flox and GR iKO mice according to a published protocol (54). Surface marker expression was analyzed by flow cytometry on a BD LSRFortessa (BD Biosciences) instrument using antibodies from eBioscience specific for the following markers: CD45 (catalog 48-0451-82), CD3 (catalog 46-0032-82), CD4 (catalog 11-0041-82), CD8 (catalog 11-0081-822), CD11b (catalog 11-0112-82), CD11c (catalog 5-0114-82), F4/80 (catalog 12-4801-82), Ly6C (catalog 17-5932-82), Ly6G (catalog 12-9668-82), and MHCII (catalog 17-5321-82). The percentages of total monocytes (Ly6C^+^), macrophages (CD11b^+^F4/80^+^), M2 macrophages (CD206^+^CD16/32^–^), neutrophils (CD11b^+^Ly6G^+^), and dendritic cells in the total CD45^+^ immune cell population were calculated.

### Data availability

The Gene Expression Omnibus accession number for the microarray data set generated with colons from PBS- or DEX-treated Flox and GR iKO mice that underwent ADX is GSE146086 (https://www.ncbi.nlm.nih.gov/geo/query/acc.cgi?acc=GSE146086). The Gene Expression Omnibus accession number for the microarray data set generated with colons from regular or DSS-containing water-treated Flox and GR iKO mice is GSE146048 (https://www.ncbi.nlm.nih.gov/geo/query/acc.cgi?acc=GSE146048).

### Statistics

Values are expressed as the mean ± SEM from at least 3 independent experiments or biological replicates, unless otherwise indicated in figure legends. For data containing 2 groups, significant differences between means were analyzed by the 2-tailed, unpaired, nonparametric Mann-Whitney test or by 2-tailed Student’s *t* test for data sets with sample sizes of less than or equal to 4. Differences were considered significant at *P* < 0.05. For data containing more than 2 groups, significant differences between means were analyzed by 2-way ANOVA test with correction for multiple comparisons. Differences were considered significant at *q* < 0.05. Statistical analysis of GR IHC staining in human colorectal tissues is detailed in *Human studies*. Statistical analysis of the microarray data sets is detailed in *Microarray study and analysis* in the Supplemental Methods.

### Study approval

The Institutional Review Board at the Fudan University Shanghai Cancer Center approved the human study protocol. All subjects provided informed consent. All animal experiments were conducted in accordance with the guidelines of the National Institute of Environmental Health Sciences/NIH Animal Care and Use Committee (approved Animal Study Proposal number 2014-0016 STL 2020).

## Author contributions

ST designed the study; performed experiments; analyzed data; designed, guided, and analyzed the clinical study; and wrote the manuscript. ZZ designed the study, performed experiments, analyzed data, and wrote the manuscript. RHO provided GR^*fl/fl*^ mice, guided the study, performed experiments, and analyzed data. WL, MJ, and LL performed animal experiments and analyzed data. XX and JLL analyzed the microarray data sets. WH, QL, and Xinxiang Li performed clinical analyses of intestinal GR in patients with colorectal cancer. QX, LC, and ASW assisted with animal studies and analyzed gene expression. JAC provided GR^*fl/fl*^ mice and guided and designed the study. Xiaoling Li guided, designed, and coordinated the study; analyzed data; and wrote the manuscript. All authors critically reviewed the manuscript.

## Supplementary Material

Supplemental data

Supplemental table 1

Supplemental table 2

## Figures and Tables

**Table 1:**
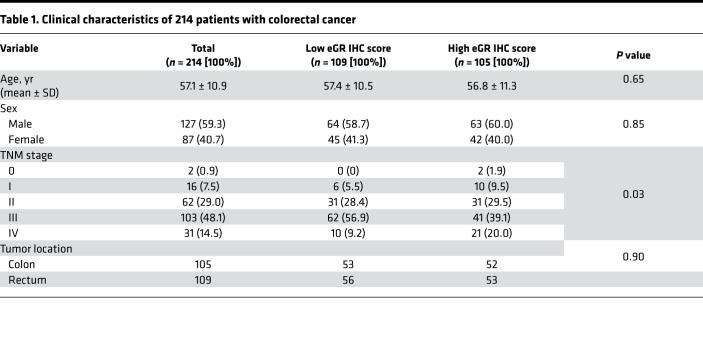
Clinical characteristics of 214 patients with colorectal cancer

**Table 2:**
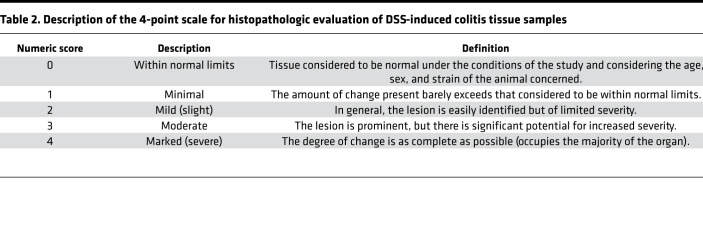
Description of the 4-point scale for histopathologic evaluation of DSS-induced colitis tissue samples

## Version 1 11/16/2021

In-Press Preview

## Version 2 12/22/2021

Electronic publication
