# Supplementary material for: Intestinal epithelial glucocorticoid receptor promotes chronic inflammation–associated colorectal cancer
Source: JCI Insight. 2021 Dec 22;6(24):e151815. doi: 10.1172/jci.insight.151815 (PMC8783679; doi:10.1172/jci.insight.151815)
Supplement: Supplemental data [file jciinsight-6-151815-s249.pdf]

Supplementary Methods

Generation of GR iKO mice

GR iKO mice (Villin-Cre^+, $GR^{flox/flox}$) and their Flox control littermates (Villin-Cre^-, $GR^{flox/flox}$), all on the C57BL/6J background, were generated by crossing mice carrying a floxed GR allele ($GR^{loxP/loxP}$)(1, 2) with Villin-Cre mice (#004586, The Jackson Laboratory, Bar Harbor, ME). Age- and sex-matched mice were used for all experiments. All mice were housed at the National Institute of Environmental Health Sciences (NIEHS) animal facility under strict specific pathogen-free conditions.

Immunohistochemistry assays

Immunohistochemical staining using anti-F4/80 (BioLegend, San Diego, CA, catalog# 123102, lot# B197365), anti-CD45 CD45 (Invitrogen catalog# 14-0451-85, Lot#4329389), anti-CD3(Abcam cat #ab5690, lot #GR317048-1), anti-Ki67 (Cat# ab16667, Lot# GR3185488-3, Abcam), and anti-GR (Cell Signaling Technology, catalog# 3660S) antibodies was performed on section at 4um thickness of formalin-fixed, paraffin-embedded colon tissues from DSS-treated Flox and GR iKO mice. Specifically, for F4/80 staining, tissues were deparaffinized in xylene and rehydrated through graded ethanol. Endogenous peroxidase activity was blocked using 3% H_2O_2, after which heat-induced epitope retrieval was performed using a 1X EDTA buffer solution (Biocare Medical, Concord, CA) in a Decloaker® pressure chamber for 5 minutes at 120 °C. Nonspecific sites were blocked with R.T.U. normal goat serum 2.5% (ImmPRESS Reagent Kit Anti-Rat Ig (peroxidase), Vector Laboratories, Inc., Burlingame, CA) for 20 minutes at room temperature. The sections were then incubated with a rat anti-mouse F4/80 monoclonal

antibody (BM8) (BioLegend, San Diego, CA, catalog# 123102, lot# B197365) at a 1:500 dilution for one hour at room temperature. Rat IgG2a isotype control serum (BioLegend, San Diego, CA, catalog #400502, lot #B126332) was used as the negative control. Then, the sections were incubated with ImmPRESS Reagent Kit Anti-Rat Ig (peroxidase) (Vector Laboratories, Inc., Burlingame, CA) for 30 minutes at room temperature. The antigen-antibody complexes were visualized using 3-diaminobenzidine (DAB) as the chromogen (DakoCytomation, Carpenteria, CA) for 6 minutes at room temperature. Finally, the sections were counterstained with hematoxylin, dehydrated through graded ethanol, cleared in xylene, and coverslipped. The percentage of the F4/80-, CD45-, CD3-, or Ki67-positive tumor area was calculated using ImageJ 2.0.0 (Fiji) software. Sections from proximal, middle, and distal colon segments from each DSS-treated Flox and GR iKO mouse were blindly quantified, and averages from at least three sections were calculated for each mouse.

Microarray study and analysis

Total RNA extracted from colon tissues of Flox and GR iKO mice was analyzed using Agilent Mouse whole-genome arrays (Agilent Technologies). To identify differentially expressed genes, ANOVA with a cutoff (FDR adjusted p-value (q-value) of <0.05) was performed using Partek Genomics Suite. Significantly differentially expressed genes (with an absolute fold change in expression > 2) were analyzed for functional enrichment with IPA software (IPA, Ingenuity Systems Inc., Redwood City, CA). Functional enrichment p-values were calculated using Fisher's exact test, and differences with a p-value < 0.05 were considered to be statistically significant. The microarray data were also subjected

to GSEA to identify potential functional enrichment. Gene sets with an FDR p-value < 0.25 were considered significantly enriched.

References:

1. Oakley RH, Ren R, Cruz-Topete D, Bird GS, Myers PH, Boyle MC, et al. Essential role of stress hormone signaling in cardiomyocytes for the prevention of heart disease. *Proc Natl Acad Sci U S A.* 2013;110(42):17035-40.
2. Oakley RH, Cruz-Topete D, He B, Foley JF, Myers PH, Xu X, et al. Cardiomyocyte glucocorticoid and mineralocorticoid receptors directly and antagonistically regulate heart disease in mice. *Sci Signal.* 2019;12(577).

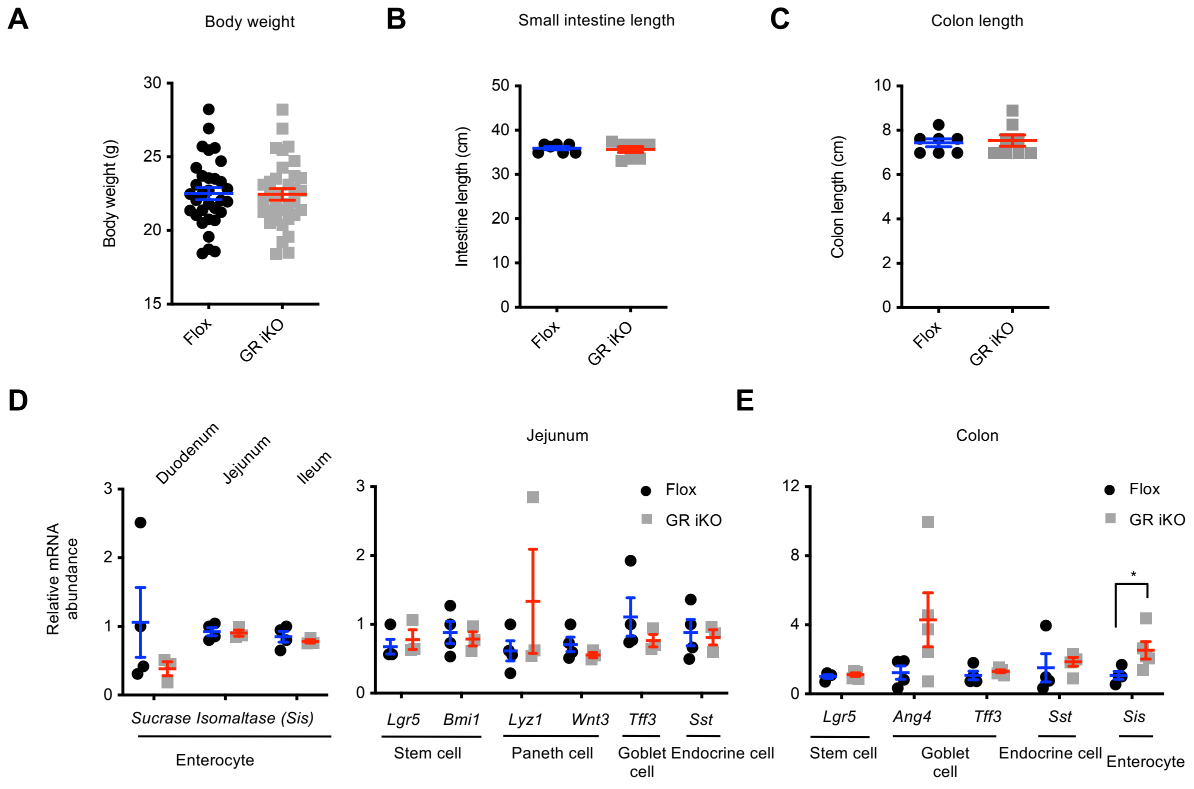

Supplementary Figure 1. GR iKO mice are phenotypically normal under normal feeding conditions.

(A) GR iKO mice have normal body weights under standard feeding conditions. Two-three-month old Flox and GR iKO mice were fed with a regular chow diet (NIH-31) (n=32 Flox and 34 GR iKO, data represent mean ± SEM). (B-C) GR iKO mice have normal length of small intestine and colon under standard feeding conditions. Two-three-month old Flox and GR iKO mice were fed with a regular chow diet (NIH-31) (n=7 Flox and 8 GR iKO, data represent mean ± SEM). (D) GR iKO mice have normal expression of intestinal epithelial markers in the small intestine. mRNA levels of indicated intestinal cell markers were analyzed by qPCR in different segments of the small intestine in Flox and GR iKO mice (n=4 Flox and 3 GR iKO, data represent mean ± SEM). (E) The expression of intestinal epithelial markers in the colon of GR iKO mice. mRNA levels of indicated intestinal cell markers were analyzed by qPCR in the colons of Flox and GR iKO mice (n=4 Flox and 5 GR iKO, data represent mean ± SEM, *p<0.05, Mann-Whitney test).

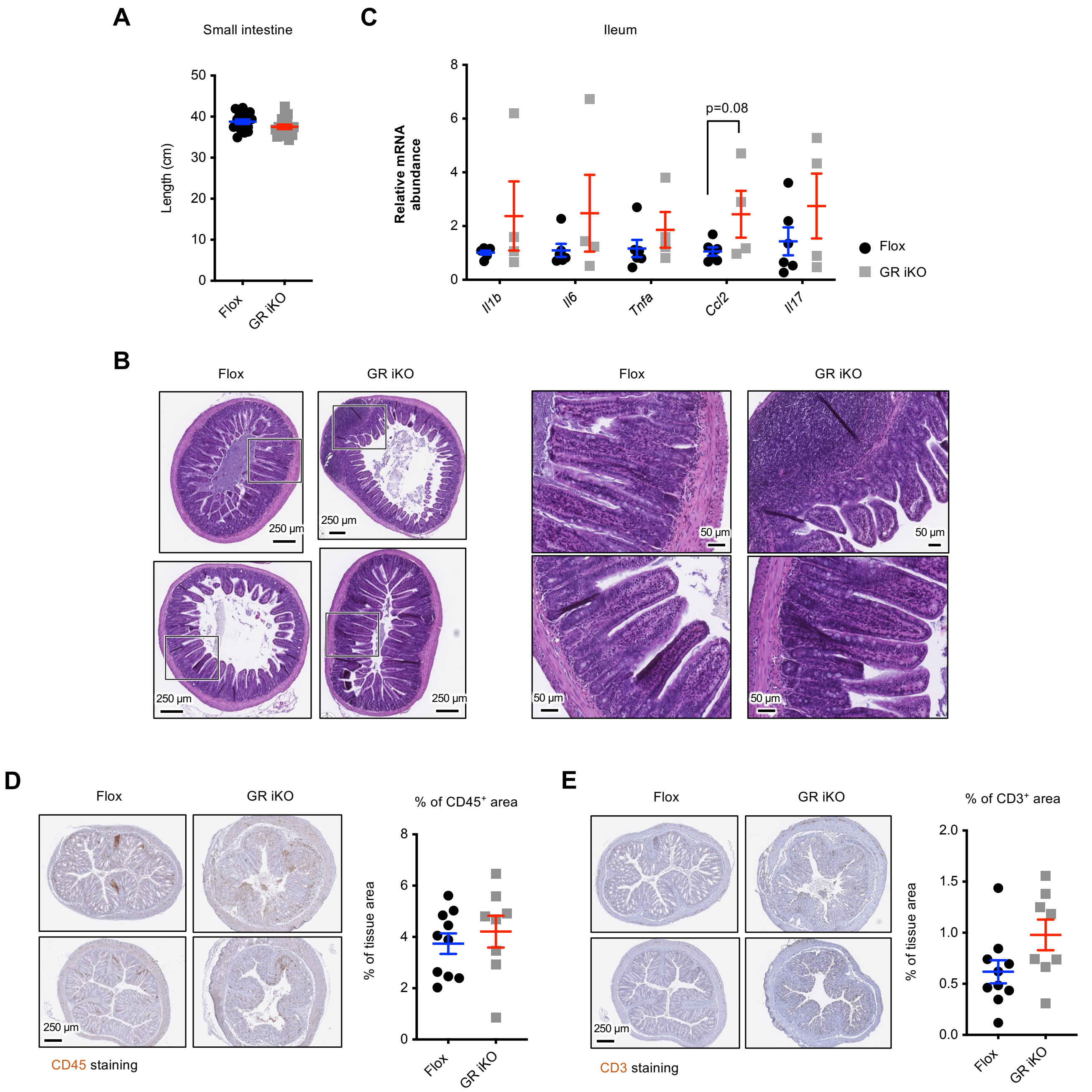

Supplementary Figure 2. Phenotypes of DSS-colitis in the small intestine and colon.

(A) GR iKO mice have normal length of the small intestines after DSS-colitis. Six-month-old Flox and GR iKO mice were treated with 2.5% DSS in the drinking water for 7 days as described in the Methods. The small intestine lengths were analyzed (n=10 Flox mice and 8 GR iKO mice, data represent mean ± SEM). (B) H&E-stained ileal sections from DSS-treated Flox and GR iKO mice. Scale bars in left panels, 200 µm; scale bars in right panels, 50 µm. (C) The expression of proinflammatory genes in the ileum of DSS treated mice (n=6 Flox mice and 4 GR iKO mice, data represent mean ± SEM). (D-E) GR-deficient colons had normal numbers of CD45-positive and CD3-positive immune cells after DSS administration. The colonic sections of DSS-treated Flox and GR iKO mice were stained with antibodies against CD45 and CD3 and quantified by ImageJ (n=10 Flox mice and 8 GR iKO mice, data represent mean ± SEM).

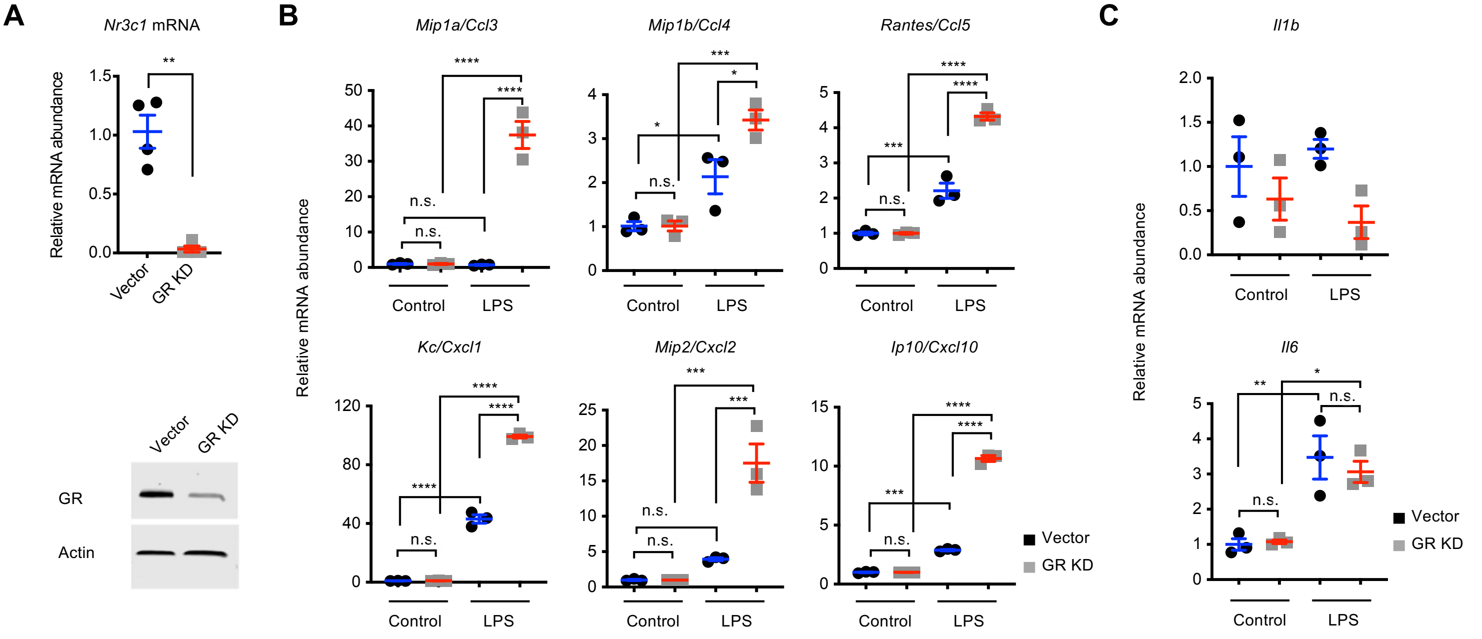

Supplementary Figure 3. Knockdown of GR in intestinal carcinoma cells increases the expression of chemokine in response to LPS treatment.

(A) Confirmation of shRNA-mediated knockdown of GR in CT26.WT mouse colon carcinoma cells. (Top) The GR mRNA levels in control cells (Vector) and GR shRNA cells (GR KD) (n=4 technical repeats, data represent mean ± SEM, **p<0.01, Student's t-test). (Bottom) The corresponding GR protein levels were analyzed by immunoblotting. (B-C) GR deficiency in CT26.WT cells increased LPS-stimulated chemokine expression. Control and GR KD CT26.WT cells were treated with 100 ng/ml LPS for 6 hours. Expression of the indicated chemokine (B) and cytokine (C) genes was analyzed by qPCR (n=3 technical repeats, data represent mean ± SEM, *q< 0.05, **q<0.01, ***q<0.001, ****q<0.0001, two-way ANOVA test).

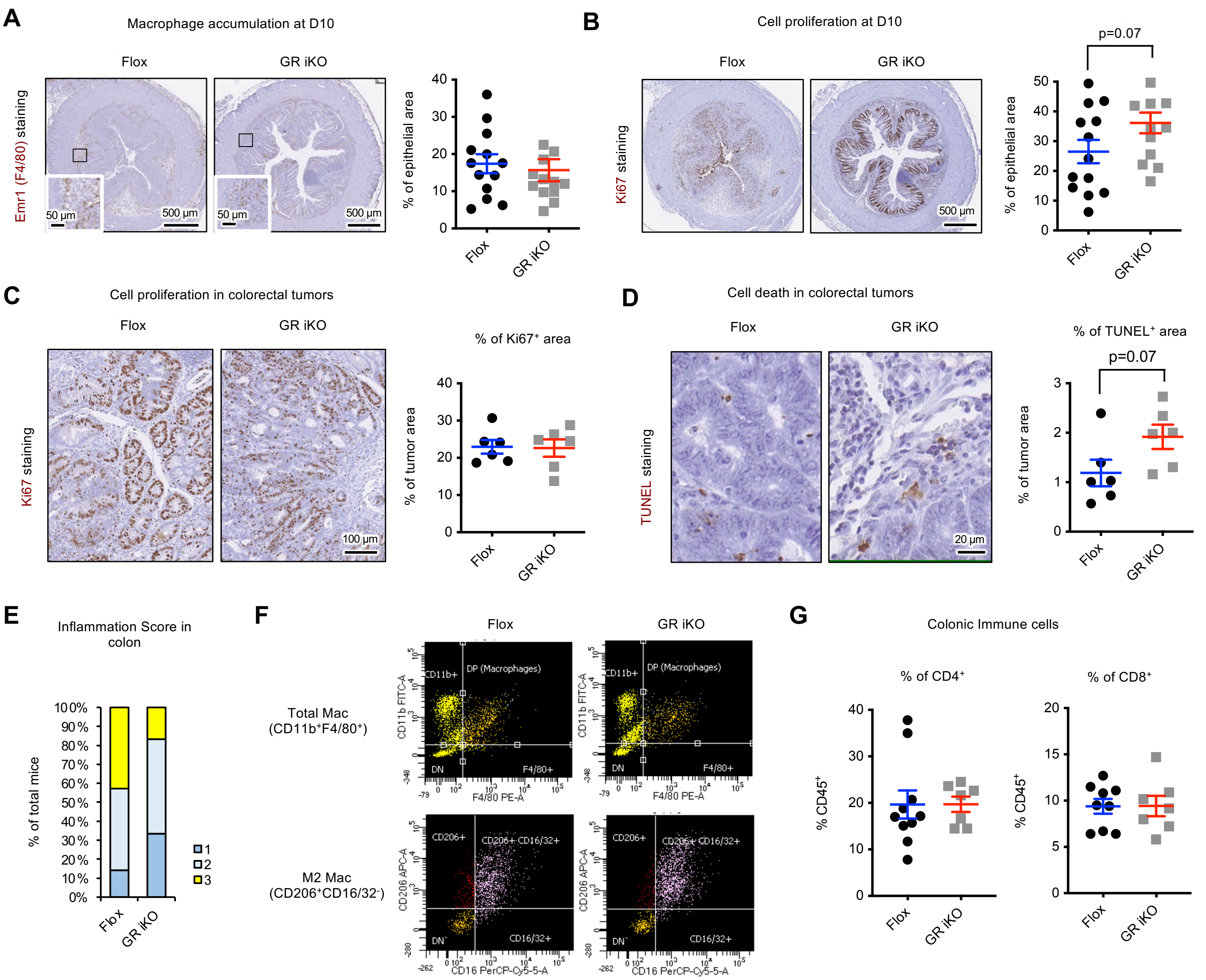

Supplementary Figure 4. GR iKO have reduced chronic inflammation associated colon cancer formation.

(A-B) Histological analysis of Flox and GR iKO mice at D10 in the AOM/DSS procedure. The percentage of F4/80 positive (A) and Ki67 positive (B) areas among colonic epithelium was quantified by ImageJ (n=12 Flox mice and 13 GR iKO mice, data represent mean ± SEM, Mann-Whitney test). (C-D) Colorectal tumors from Flox and GR iKO mice have comparable proliferation and cell death. Colorectal sections from Flox and GR iKO mice in the AOM/DSS colorectal cancer model were stained for Ki67 and TUNEL as described in Methods. The percentage of Ki67 positive or TUNEL positive areas among analyzed tumor area was quantified by ImageJ (n=6 mice for each genotype, each data point represents an average of at least two independent colorectal tumors from one mouse, data represent mean ± SEM, Mann-Whitney test). (E) The inflammation from the H&E colonic sections at the final tumor stage were evaluated and scored as described in Methods (n=8 Flox and n=8 GR iKO mice). (F) Representative FACS plots for colonic total macrophages and M2 macrophages. (G) T cells in the colon of Flox and GR iKO mice at the end stage of AOM/DSS colon cancer model. The percentage of $CD4^+$ T cells and $CD8^+$ T cells among total $CD45^+$ immune cells were analyzed by FACS as described in Methods (n=20 Flox and n=14 GR iKO mice, colon tissues from 2 experimental mice were used for each data point, data represent mean ± SEM).

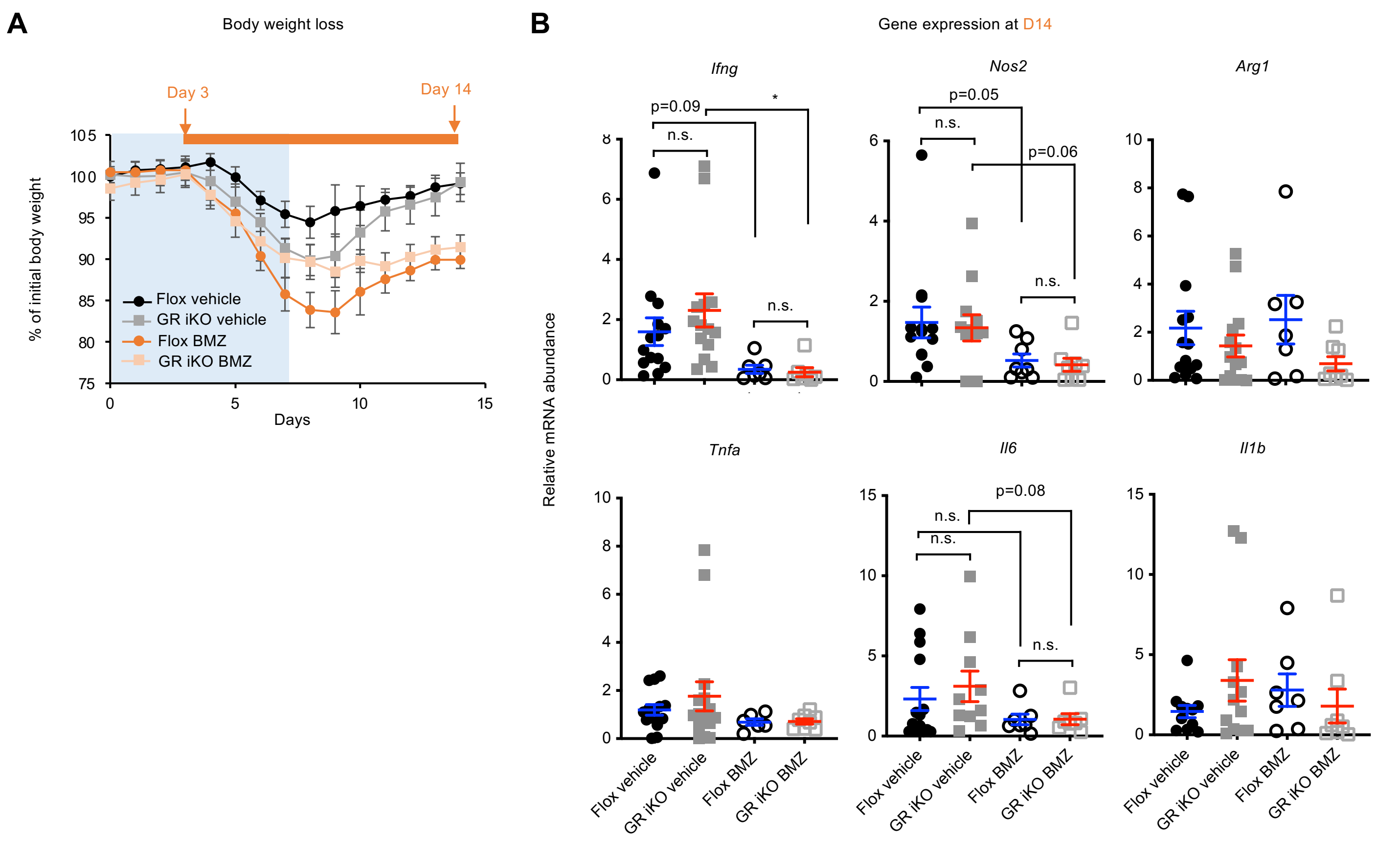

Supplementary Figure 5. Short-term impacts of early-phase BMZ treatment on the expression of inflammatory genes and immune regulators in the colons in the AOM-DSS colon cancer model.

(A) The body weight of Flox and GR iKO mice during a short-term AOM/DSS treatment with or without BMZ. Flox and GR iKO mice were injected with one dose of 8 mg/kg AOM, followed by 7 days treatment with 2.5% DSS in the drinking water (light blue area) and recovery for 7 days. During D3 to D14, they were subjected to oral treatment of either vehicle (0.125% ethanol) or 2.5 µg/ml BMZ in 0.125% ethanol (n=5 Flox vehicle, 7 GR iKO vehicle, 7 Flox BMZ, and 9 GR iKO BMZ, data represent mean ± SEM). (B) The expression of indicated proinflammatory and immune genes in D14 was analyzed by qPCR (n=14 Flox vehicle, 14 GR iKO vehicle, 7 Flox BMZ, and 7 GR iKO BMZ, data represent mean ± SEM, *q<0.05, two-way ANOVA test).

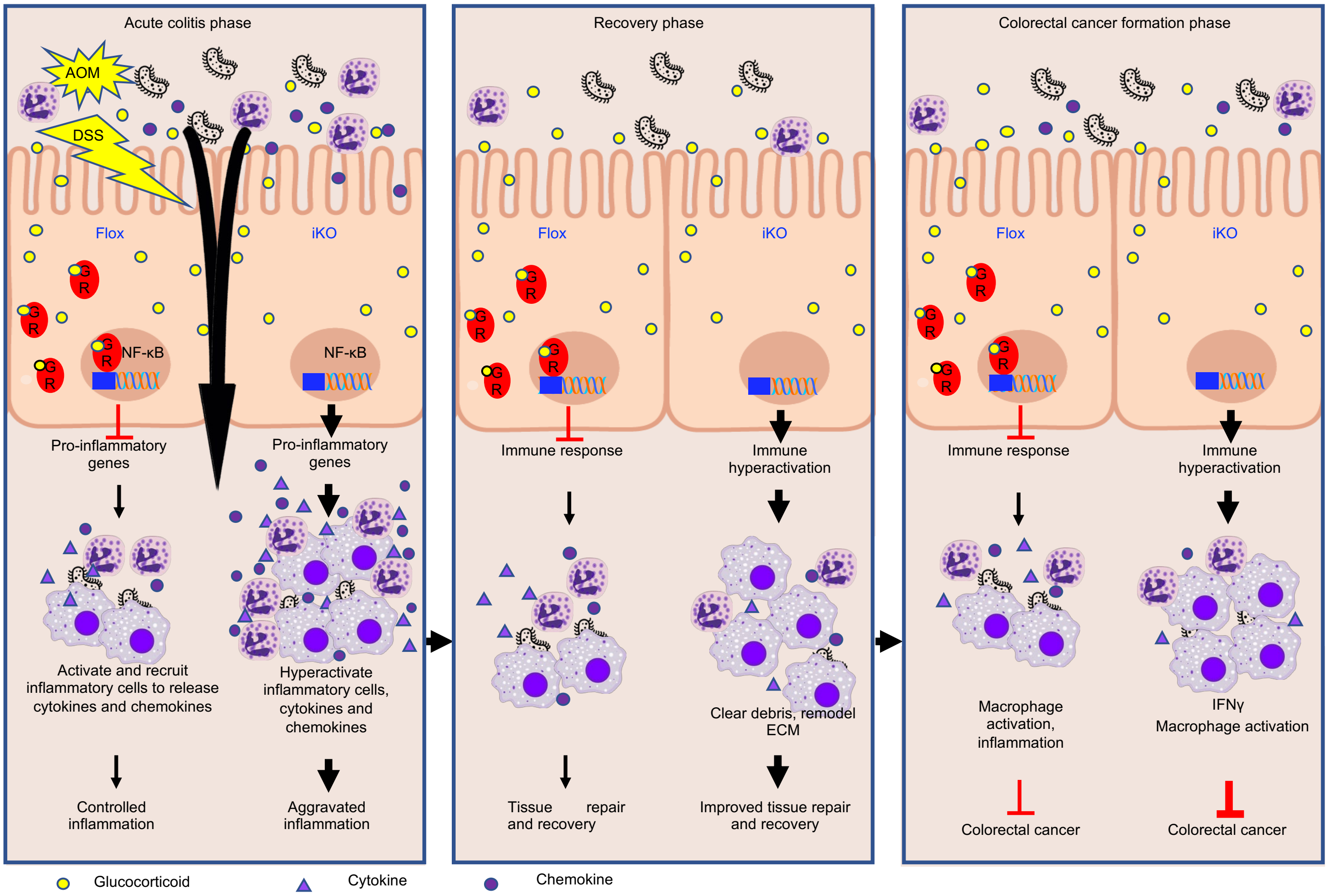

Supplementary Figure 6. Intestinal-Epithelial GR signaling suppresses acute colitis but promotes chronic inflammation-associated colorectal cancer.

intestinal-epithelial GR signaling reduces intestinal inflammation and immune activation in response to acute chemical-induced colitis. However, this endogenous intestinal-epithelial GR-mediated repression of inflammation and macrophage activation in the early-phase of chronic inflammation delays tissue repair in the recovery phase, resulting in increased formation of colorectal cancer at a later phase.
